# Science Outreach: Providing an Authentic Independent Research Opportunity in Materials Science to School Students

**DOI:** 10.1021/acs.jchemed.5c00697

**Published:** 2026-05-18

**Authors:** Neil Garrido, Andrew J. Lee, Clare Turnbull, Paolo Actis, Alison Rouncefield-Swales

**Affiliations:** † Institute for Research in Schools, London, 165 Queen’s Gate, London, U.K. SW7 5HD; ‡ Bragg Centre for Materials Research, 4468 University of Leeds, Woodhouse Lane, Leeds, West Yorkshire, U.K. LS2 9JT; § School of Electronic and Electrical Engineering, 4468 University of Leeds, Woodhouse Lane, Leeds, U.K. LS2 9JT

**Keywords:** High school, Outreach, Inquiry-based, Materials science; Independent research project, DNA origami

## Abstract

Through the emerging field of bio-nanotechnology, students from UK high schools were provided with an opportunity to explore the use of DNA as a self-assembly building material to create unique DNA origami designs and evaluate these using web-based software. The project helped widen awareness of the cutting-edge research within materials science, enabled the participants to work with a high degree of independence, and reinforced the practical techniques required to synthesize a predesigned origami structure at school. This article describes the results of a study of this long-term, student-led independent research project. We collected data from 51 young people aged 16–19 engaged in the project using group interviews and found authentic research experiences can foster greater personal autonomy, sustain engagement, and be used to improve the perception of subjects such as materials science. The project also gave participants a greater understanding of the broader scene relating to careers within STEM and research as well as improved research skills through thinking and acting like a researcher.

## Graphical abstract

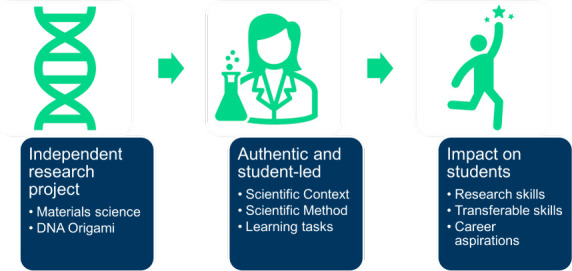

## Introduction

Despite its importance within the broader economy, young people often overlook materials science careers leading to a shortage of skills across the profession.^1^ In part, this is exacerbated by the limited inclusion of materials science within the the National Curriculum for England.^2^ Materials science within the science curriculum focuses on a limited number of materials and their properties including: ceramics, plastics, and metals. This overlooks the significance of this scientific discipline by ignoring recent advances and extensive research being conducted on novel materials. To compound this problem, a report published by the Institute for Materials, Minerals, and Mining^3^ shares that students have limited awareness and understanding about materials science and are unaware of the subjects necessary to pursue materials science at the university level. Consequently, activities that enhance the taught curriculum could offer valuable opportunities for students and teachers to gain a distinct perspective on materials science.

Within the UK education sector, outreach and enrichment initiatives led by universities, educational organizations, and other institutions have attempted to provide young people with opportunities to experience STEM beyond the taught curriculum, to engage students in science, increase motivation, improve attitudes, and broaden the talent pool. Although efforts to increase young people’s engagement in science should be encouraged, the overall impact of these activities is less clear, and it remains uncertain if certain methods are more effective than others. The Institute for Research in Schools (IRIS) approach is to provide young people in schools and colleges with access to an authentic student-led research experience. Our main research interest in the present study is to find out whether participating in an authentic research experience can improve students’ research and transferable skills, as well as increase their aspirations toward scientific careers.

## Literature Review

### Independent Research Projects (IRPs) and the Role of Authenticity

In recent years, collaborations between universities and industry have given rise to initiatives that allow students, typically aged between 11 and 18, to engage in longer-term research investigations.^4–7^ Two similar initiatives, ORBYTS^8^ and PRiSE,^9^ while small in scale and solely focused on physics, offer students the opportunity to undertake IRPs and are contributing to broadening access to longer-term research experiences for school students across the UK.

IRPs actively engage students in a research cycle, that is typically student-led, open-ended, supported by a teacher or researcher and provides substantial autonomy.^7,10^ There is growing evidence that these initiatives have a greater impact on young people’s interests and aspirations compared to traditional one-off activities.^11^ School students in the UK, particularly in England, have limited chances to participate in scientific research and interact with science in a manner akin to that of researchers and scientists. IRPs offer an essential avenue for students to gain invaluable experience grounded in the practices of professional scientists.^12^

One aspect of IRPs that has received little attention is whether they provide an authentic experience of scientific research. The concept of authenticity, although prevalent in educational discourse, is nuanced and frequently applied in an inconsistent manner.^13^ A multidimensional model^14^ emphasizes that authenticity in science education can be achieved through combining external aspects of ‘real-world’ and ‘disciplinary’ authenticity with the internal aspect of ‘personal’ authenticity. This dual recognition of the external and internal perspectives incorporates the viewpoint of the educator as well as the individual perspective of the students. To achieve ‘real-world’ and ‘disciplinary’ authenticity, experiences should mirror the activities undertaken by scientists and researchers^15^ and enable students to collaborate, utilize discipline-related techniques or processes,^16^ and engage with each stage of the research cycle.^17^ To foster ‘personal’ authenticity, it is essential that students perceive the research as genuine, recognize their contributions as relevant, and feel that the project provides meaningful learning opportunities.^18,19^

Previous studies on the role of authenticity in science education have shown that authentic learning can trigger a positive motivational response and foster a sense of ownership and commitment, aiding students in mastering scientific and transferable skills.^16^ Additionally, evidence indicates that authentic learning experiences can enhance student engagement^15,20^ while improving knowledge, self-efficacy, and career aspirations.^15^ However, there is little research into the importance and impact of authenticity in IRPs.

### Designing DNA Origami: An IRP Using Computer-Aided Design

DNA origami is an emerging field within bio-nanotechnology and focuses on using biological building blocks for engineering solutions and technological advancements.^21^ It was first demonstrated that DNA could be used to construct nanostructures in the early 1980s.^22^ Since then, research into constructing nanomaterials using DNA has grown significantly with the term ‘DNA origami’ used to showcase the versatility of DNA in self-assembly by creating various nanostructures.^23^ The principle is relatively simple: a single strand of DNA (ssDNA) acts as a scaffold folded into a 2D shape (Figure 1) which is then fixed by short complementary ssDNA staples, forming immobile Holliday junctions (Figure 2). The synthesis process involves heating and slowly cooling the DNA, allowing nanoscale structures to self-assemble with significant accuracy. It is a powerful tool which enables the creation of nanoengineered materials with tailored properties resulting in wide potential for applications including medicine,^24^ data storage,^25^ nanorobots,^26^ and more complex 3D designs.^27^ The synthesis itself requires few hazardous chemicals and is a relatively straightforward process, meaning it can be done in a school setting.

**Figure 1:**
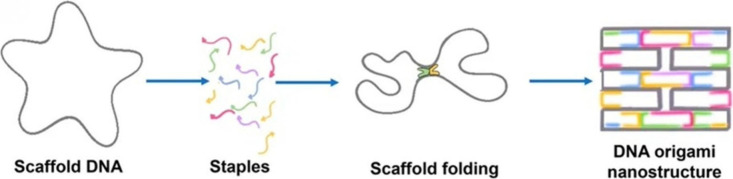
DNA origami assembly from the directed folding of the scaffold strand (gray) by using complementary staple strands (multicolored).^31^ Licensed under CC BY 4.0.

**Figure 2:**
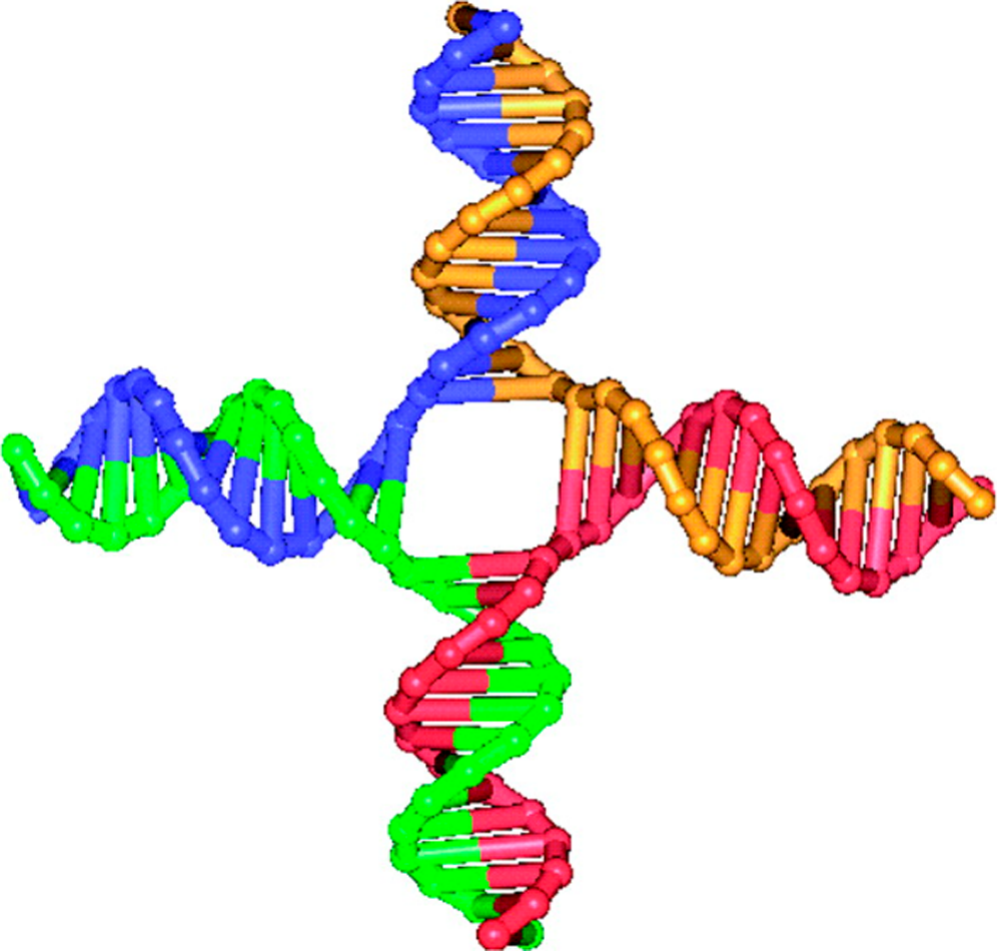
Creation of immobile Holliday Junctions^32^ enables staples to bind the scaffold into the predetermined shape. Copyright 2000 National Academy of Sciences, U.S.A.

DNA origami can act as a scaffold for assembling inorganic nanomaterials to allow precise spatial organization, which is crucial for developing functional materials.^28^ It enables the construction of enzyme-based nanodevices, biological nanosensors, and drug delivery systems, allowing controlled release and targeted delivery.^29^ Recent research shows how DNA origami can be used to build reconfigurable nanostructures, molecular motors, and force sensors, opening doors to smart materials that respond to environmental stimuli.^30^

The process of creating DNA origami designs has been simplified by the development of computer-aided design software. This advancement allows researchers to construct intricate DNA origami structures with relative ease.^33^ caDNAno, an open-source software package, was created to simplify DNA origami design by automating calculations needed for the creation of the staple sequences which would ensure a high success rate of folding DNA into desired shapes. It is effective in generating both 2D and 3D structures.^33^ Creating simple 2D designs requires only basic knowledge of the software and DNA structure and, as such, was deemed suitable for high school students. There is some evidence that computer aided design (CAD) tools can support integrated science learning with a design process.^34^

## Evaluating Designs Using CanDo

While caDNAno is a useful tool to help design DNA origami structures, interpreting its graphical representations can be challenging. For instance, gaps between helices are not to scale, potentially leading to misconceptions about the overall structure of the designs. To address this issue, training carried out with students included additional software capable of converting caDNAno designs into atomic models, images, and movies. This approach allowed students to visualize their designs in 3D, helping them identify limitations. A common early design flaw was failing to account for the helical nature of DNA, resulting in designs that appeared flat in caDNAno but exhibited significant twists when modeled. This prompted participants to understand the reasons behind these distortions and modify their designs accordingly. The software known as CanDo^35,36^ can be used to evaluate models created in caDNAno.^35^ By incorporating CanDo, students gained insights into the stability of their designs and accessed images and movies of the atomic structure, enhancing their understanding of abstract concepts to more familiar visual representations. Additionally, file formats exported from CanDo allowed students to use molecular modeling software for further exploration of their designs.

### DNA Origami as a Model for Authenticity in IRPs

The objective of the DNA Origami IRP was to provide students with an authentic research experience in materials science, enabling them to design, synthesize, and evaluate DNA-based nanostructures using computer-aided tools and laboratory techniques. The outline of the project attempted to create a highly realistic research environment by giving them insights into scientists’ work and access to personal expertise through the team at the Bragg Centre for Materials Research at the University of Leeds. The structure of the project is based on a model developed by the Institute for Research in Schools whereby students progress from learning foundational concepts to conducting their own research and presenting their findings:

- Phase 1: Preparation and launch (focused on preparing for the project)
- Phase 2: Background research and skills development
- Phase 3: Student research (a sample of atomic force images of the DNA origami assembled by students is provided in the : Atomic force images of the DNA origami assembled by the students (PDF))
- Phase 4: Artifact development and science communication (a sample of student posters are provided in the : A small sample of student posters with all names redacted (PDF))

This model has been refined over the past decade, based on our extensive experience working with schools and universities as well as direct feedback from students and teachers. The overview of the pilot project which was shared with schools is provided in the : DNA Origami pilot project overview (DOC).

Aligning with the authenticity model,^14^ the DNA Origami project intends to achieve ‘real-world’ authenticity by offering content that is both relevant and topical. It provides students with access to a highly realistic research environment and gives them insights into scientists’ work and access to personal expertise through the team at the Bragg Centre. Students must dedicate significant time to their projects, as the open-ended nature of the approach necessitates investigation, problem-solving, decision-making, and reflective learning. Due to the support of the university partner, participating students are invited to a university symposium to gain an insight into the context as well as the work of researchers in the field. Teachers are provided with resources and training to guide science content supplemented by direct engagement with university researchers. In addition, students are provided with support from the university researchers, IRIS staff, as well as their own teachers. The model aims to ensure that teachers and students learn alongside each other, fostering a collaborative learning environment. Fulfilling requirements relating to ‘disciplinary’ authenticity, students receive training in university-standard techniques and use equipment used by real researchers, enabling them to engage in genuine scientific enquiry and help them to develop technical skills such as serial dilutions and pipetting, Ultimately, students are given the opportunity to present and discuss their research in a conference setting, exposing them to a further critical research activity.

The third dimension ‘personal authenticity’ relates to students’ perception of authenticity throughout the whole project. Personal authenticity is difficult to build in to project design due to it being related to the student’s experience—we cannot assume that a learning environment designed to be authentic is also perceived as such by learners. However, we can consider the students’ perception of their experience of the IRP and whether they feel this to be authentic.

## Methods

### School Profiles

51 students from five nonselective state academies or secondary schools took part in the pilot project (Table 1). All pilot schools had a higher proportion of students receiving free school meals (FSM6) than the national average, which serves as a useful proxy for disadvantage. This highlights the project’s reach into schools serving more disadvantaged populations. The pilot commenced in early 2021 and spanned approximately nine months. Students were encouraged to take part by teachers and open invitations, and all were studying at least one A Level science subject. To assess the project’s adaptability, these schools represented a range of previous experience levels from those with no prior research involvement to those with a well-established research culture. Teachers participated in preparatory meetings and received resources and ongoing support from IRIS and the Bragg Centre. Following the pilot project, 689 students participated in this project. IRIS started gathering gender data on student participants in 2021. Of the 465 students with available information, 60.5% of DNA Origami participants have been female (see : DNA Origami project student participation and survey data (DOC)).

**Table 1:** Pilot School Profiles, 2019 ^37^

school	location	FSM6^a^	no. of students
A	West Midlands	50.5%	8
B	North West	57.3%	10
C	West Sussex	28.4%	14
D	West Yorkshire	24.8%	7
E	South Yorkshire	18.2%	12

a Pupils who have been eligible for free school meals (FSM) at any point in the preceding six years. In 2020, the national average was 15.4%

### Safety Statement

The DNA synthesis is a relatively straightforward process to complete in a school setting and no unexpected or unusually high safety hazards were encountered.

### Data Collection and Analysis

Qualitative data were gathered throughout the pilot year of the project, with the findings informing the project’s future development. This work was led by the IRIS research and evaluation team and was conducted via a combination of the following methods:

- 19 student questionnaire responses
- focus groups with 11 male and six female students
- interviews with six teachers/technicians at partner schools

The survey consisted of open questions to enable participants to share their experience and closed questions to explore the impact on students and to stimulate their reflection on their experience. The survey data were analyzed using SPSSv14 and descriptive statistics reported with the open text being thematically coded. Quantitative results from the pilot year student questionnaire and analysis of questionnaire responses from the project in 2023/24 and 2024/25 are provided in : DNA Origami project student participation and survey data (DOC). The questionnaires in the pilot year were exploratory and offered limited statistical insights; findings from the pilot project have led to a more refined and systematic data collection approach in subsequent years.

Individual interviews with school staff were conducted online using MS Teams with the purpose of allowing respondents to speak openly and freely to uncover new insights.^38^ The teacher interviews explored participation in the project, the perceived benefits to themselves and the students, and any difficulties they had experienced in delivering the project. The student focus groups took place face-to-face during a symposium at the Bragg Centre and focused on the students’ participation in the project, the perceived benefits to themselves, as well as any challenges they had experienced.

Interviews were conducted by two interviewers, took between 30 and 60 min, and were recorded and transcribed verbatim. Interview transcripts were analyzed by two researchers using a five-phase process to explore and understand the data.^39^

1. Organizing
2. Sorting
3. Understanding
4. Interpreting
5. Explaining

### Ethics

Ethical standards were upheld by adhering to the guidance of the British Educational Research Association Ethical Guidelines for Educational Research^40^ in relation to informed consent. As such, there was verbal communication to outline the aims, and a clear explanation of participation was voluntary. Participants were informed of their right to withdraw and that all data would be anonymized and kept secure. The act of questionnaire completion was noncompulsory.

## Findings

In this section, we draw on feedback from participating students and teachers to evaluate the experience of involvement in the project using data from the surveys as well as from interviews and focus groups. In constructing the themes, we identified three broad categories of codes. The first focuses on the meta-theme which centers around ‘the importance of personal authenticity’ and explores the students’ actual perception of the research project. The two subthemes connect students’ perception and experience of authenticity across the process and its impact on them. These subthemes reflect the participants’ perceptions of the personal gains and outcomes as a result of their own investment in the project.

### Meta-Theme: The Importance of Personal Authenticity

The meta-theme of personal authenticity was common across all interviews and focus groups. This theme was interwoven throughout the discussions about the scientific context and methodology as well as the student experience and impact.

## Authenticity of Context

Students were enthusiastic about participating in a research project that they considered topical, relevant, and of broad applicability. The context not only made the research project itself more engaging and relevant but also contributed to a sense of involvement in genuine scientific activities. This personal authenticity gave rise to feeling like “real scientists”, which enhanced student engagement, motivation, and their overall learning experience. As one student explained:

Science is about breaking into new ground, going into the unknown, looking to the unknown but then also backing that up with evidence and trying to prove the unknown...did I feel like a scientist? I did because I’m looking at stuff which I had no idea about and then also acting on it as well. (Year 12 male student, School C)

The project gave students insight into a real-world context. Students acknowledged the scientific importance of DNA origami and its potential applications in various fields. This holistic impression of DNA, beyond just coding bases and storing genetic information, increased engagement because students could see the potential impact of the work, such as the curing of diseases like cancer. The experience also provided them with diverse and interdisciplinary perspectives. As their understanding expanded over the course of the project, students developed a greater personal connection with the project. As one student elaborated:

It provided me with real world issues and context to what I’m learning. To learn about DNA you just see it as a material that is just there to code DNA and code bases and store genetic information, but it gives you this more holistic impression about how it provides a more overarching thing. I think it also made it more engaging because you can see eventually that your work might actually have some effect, like it can be used to cure things like cancer and other issues. (Year 12 male student, School C)

## Authenticity of Method

Students greatly appreciated the opportunity to master advanced techniques that surpass the resources typically available to them in school. This practical application of science allowed students to experiment and analyze results in ways they would never experience in a typical classroom setting. They valued the chance to develop skills using high-quality, university-level equipment that their schools did not have access to.

It is something that we have never done before, and it is something that we have never heard of. It is the first time we’re getting to use certain types of equipment, like actually having pipettes. I know we use really rubbish plastic ones, but these are real micropipettes. Then when [researcher] came in, we did the whole centrifuge, and I really enjoyed that. (Year 12 female student, School D)

I think it has been really useful to look at this practical application. We do a lot of sitting in class and reading from a textbook so getting to actually get up, and pipette things and use a centrifuge, or getting to actually use these systems and then analyze the results, like not just creating a structure on caDNAno but actually looking at the stability of it on CanDo stuff like that. You would never get that experience in a classroom (Year 12 female student, School E)

## Authenticity of Learning Tasks

Students felt ownership of the scientific process and the success of their project. They found that while there were challenges and obstacles, collaborating with peers to investigate a real-life issue was motivating and inspiring:

I think it is satisfaction. It is seeing a question answered in a way that you feel is as complete as it can be. It is the whole thing of having to keep working at it until one can find a way to make it function. It is so iterative, you have to keep working at it, and it is like an itch that you just keep scratching, you have to answer this question, and that was really cool. (Year 12 female student, School B)

Presenting their research at a conference or symposium solidified the research cycle and provided closure to the project. It instilled in the students a feeling of expertise regarding their work as well as enhancing their ability to communicate scientific findings to a broader audience.

I think the best bit is making a poster; researching it and developing your skills, working with everybody, sharing information, working with [IRIS] and working with everyone, and then just coming here and getting it printed, working on a poster which you can then present to other people. (Year 12 male student, School C)

The teachers recognized the independence granted to the students by the project. They provided guidance, fostering a unique teacher-student relationship, and cultivating a community within the schools that is engaged in science.

It just feels like we’ve got a real community.... It is like, we talk science, and we have all these little science things; genuinely, we have all these really keen students, and it is just feels like, I’m part of something and it makes me want to stay. (Teacher, School E)

He [teacher] was always there for help, but I think we were in control; it was definitely OUR project. He [teacher] was learning alongside us, so he could be involved, and I think he enjoyed learning it as well. (Year 12 students, School E)

### Subtheme 1: Developing Scientific, Research, and Transferable Skills

The first subtheme elucidates the students’ perceptions of the enhancement of their scientific, research, and transferable skills. Students reported enjoying the research process and reflected on how various skills had improved through their participation.

## Working Together

Students discussed how collaborating within teams throughout the research process significantly enhanced their skills, such as communication, problem-solving, and cooperation. The teamwork allowed the students to value individual contributions and learn from one another. These experiences provided valuable material for future employment, university applications, and interviews, showcasing their ability to work both independently and as part of a team. Overall, the teamwork component was seen as highly beneficial and educational, with collaborative efforts deemed crucial for skill development and essential to the project’s success. This sentiment is exemplified by two students:

The skill of sort of teamwork and being able to bounce off of other people are definitely things that is useful. The way we learned to all collaborate with each other, using everyone’s individual skills, will definitely come in useful. (Female, Year 12 students, School E)

The best part was teamwork, being able to get a team of five students to work together after school, which was one of the best things. The research, being able to delve into some deep science and be able to see the applications of it, that is one of the others and last, presentation skills.... Being able to talk...about our project that was one of the biggest skills we could have learned.... I think for me it is one of the most revolutionary like projects I have ever taken part in. (Year 12, Male student, School D)

The emphasis on working as a group and the collaborative nature of the project ensured that neither the teacher nor any individual student was considered an expert; instead, both students and teachers approached the task as a collective learning experience. This helped to foster a positive and supportive atmosphere:

It was the idea that no one was the resident expert. We were all learning it together, even if [the teacher] had never seen it before, so we were all approaching the software together as a new thing for everyone, and it made for a really good atmosphere. (Year 12 male student, School E)

Teachers observed the evolution of group dynamics as students took the initiative to support and, where necessary, teach each other how to master software or scientific techniques. These interactions helped form positive group dynamics as students became more competent and fed into levels of engagement, as illustrated by the following teacher:

Problem-solving and working together in groups. I’ve never seen students work so effectively together in a group, to be honest. It was phenomenal. They just genuinely got stuck in. I think that kind of agency, and they felt like they were quite important. (Teacher, School E)

The project’s student-led nature further empowered the participants’ ownership of their work. While teachers and other staff were available to support and guide students, the work was driven entirely by the students. This autonomy underscored students’ capability to lead and execute complex tasks. As illustrated by one student interaction:

It was very student-led. Obviously, there were contributions from [IRIS] and [teacher] helping us, not writing it for us but giving us recommendations, proofreading it for example, but nothing in terms of... (Male 1)

Writing the content...it was all of us... (Female 1)

Yeah exactly, it was all student-led... (Male 2) (Year 12 students, School D)

Overall, the group work involved in the research project was pivotal in fostering a range of competencies, including technical proficiency, teamwork, time management, and leadership. These experiences not only helped to prepare the students for future academic and professional endeavors but also instilled a strong sense of confidence and agency in their abilities.

## Scientific Skills

Students acquired hands-on experience with advanced scientific equipment such as Eppendorf research pipettes and centrifuges, which are typically unavailable in their school settings. This practical exposure facilitated a deeper understanding of the processes involved in preparing solutions and working with various concentrations. As discussed by one student:

One of the main things we learned to do was pipette with actual pipettes...to be able to use these real pipettes and different sizes as well, so that was really interesting to learn how to actually pipette and that process. Another thing that I really enjoyed was learning how to centrifuge and also make up all of the stock solutions. One of the key things that we had to do was calculate the amount of each solution that we needed, and that was quite fun because it really tested our problem-solving skills, but it was also just enjoyable to see that process and to see what work goes into making the solutions and all the different concentrations. (Year 12 male student, School C)

The project fostered an appreciation of the complexity and precision required in scientific research. Students learned the importance of accurate measurements and the extensive processes involved in research. This experience also provided students with a realistic perspective on research, emphasizing the time and effort required for the distinct tasks.

## Solving Complex Problems

The development of problem-solving skills was identified as a crucial competency; these skills were necessary not only for scientific processes but also for technological aspects of the project.

It was very much: we had a problem, we solved it, and we got onto the next thing. It was very much learning it as we go along. (Year 12 male student, School E)

Because of their computing prowess, they solved lots of problems. So, there’s lots of problem solving that they were getting to do that I did not realize they’d have to do.... That was just really enjoyable (Teacher, School E)

### Subtheme 2: Stimulating Aspirations

The second subtheme refers to how the project stimulated students’ aspirations for future scientific and research careers. Students were inspired by learning about advancements in these technologies and their real-world applications.

I think before going into this [I thought] material science was concrete and metals, it is really interesting seeing all the other different angles, like all the nanotechnology and all this stuff.... It is a lot more interesting than I originally perceived and the DNA stuff, as well. With our research, we saw the software first and saw what we could do, but then in our research we looked deeper into the applications, and it is so much more expansive than I originally thought. (Year 12 male student, School E)

Through participating in the project, students gained an increased understanding of and interest in science-based careers, alongside gaining valuable insight into research and careers that extend beyond traditional classroom learning.

The whole project is different to the studying in college or GCSE and if people are looking into going into science, doing a project like this will give you good insight into what actually goes on. I think that more projects like this would encourage more people to go into science. Look at the stuff we’ve just done, doing the origami and what people are trying to use it for, and that will give a lot of motivation and inspiration to people to actually go into the field. (Year 12 male student, School C)

The experience brought them into contact with a field of study beyond what they had experienced at school and helped to expose students to the vastness of scientific research. This provides students with a more realistic view of research, including the time and effort, as well as the multidisciplinary nature of many scientific advances.

They [students] do not think about the multidisciplinary, interconnectedness of subjects.... I think the idea that you can have material scientists and engineers that are using molecules from biology and applying some principles from biology. I think that is one of the main benefits for them.... I think it is been a brilliant project for that. I love projects where students get to see that scientists do not work in isolation from each other, and that it requires a range of expertise to do well in those settings. (Teacher, School B)

Through exposure to researchers in the field, students gained a greater appreciation of career opportunities in materials science and more broadly across scientific research. Moreover, the project inspired students to consider science and research careers more seriously.

## Discussion

The DNA Origami IRP in materials science successfully integrated real-world, disciplinary, and personal authenticity.^16^ Compared with traditional outreach activities, IRPs offer greater depth and autonomy, allowing students to engage in the full research cycle. While other initiatives may spark interest, IRPs are distinguished by their emphasis on authenticity and student-led inquiry. While students may have access to other science outreach activities, we would suggest that IRPs complement these by providing a unique opportunity for independent research and deeper engagement with scientific practice.

By drawing on a contemporary scientific field, advanced scientific methods, and research processes, the project supported students in developing knowledge, skills, and aspirations. The study found that students perceived there to be authenticity in the context, method, and learning tasks, which contributed to the development of their skills and aspirations. In prioritizing authenticity, this IRP provided students with a realistic and meaningful experience of scientific research, which was highly motivating. Participating in an authentic research experience allows students to gain a deeper understanding of scientific concepts and processes while working collaboratively. Students showed strong ownership of their research and a commitment to their teams, gaining valuable knowledge and experience from their participation. Students valued how the project helped them to feel that they had crossed a conceptual threshold in becoming researchers. This included devising research questions, collecting and interpreting data, engaging with advanced techniques, and communicating their work with peers and experts. Although the project presented challenges, students appreciated the responsibility bestowed upon them and valued the agency they had been given. The experience had a positive influence on students’ future career aspirations, and they reported feeling better informed about opportunities in materials science and scientific research. If science can be made more authentic for students, it can increase their motivation and interest.^13,15^ However, we recognize that various elements could impact levels of student engagement and project outcomes including, but not limited to, their teacher’s attitudes and comfort in managing uncertainty as well as variation in levels of support.

The IRIS model has demonstrated scalability, with over 689 students participating in this project since the pilot covering a broad geographic spread and a large number of schools. Key factors supporting scalability include the provision of online resources and tutorials, teacher training, partnerships with universities, and a move to utilizing web-based software to improve accessibility. However, expansion may be limited by access to laboratory facilities and researcher support.

## Conclusion

The DNA Origami project successfully achieved personal authenticity for students by integrating the real world and disciplinary authenticity. The study found that the DNA Origami research project provided students with a positive experience, leading to a greater appreciation of science beyond what they encountered in the classroom. The project resulted in improvements to a range of transferable and research skills, and there is evidence to suggest that students are more likely to consider careers in science and research. These improvements can be attributed to the authentic, rewarding, and motivating experience.

While the positive impacts of the project are clear, the design of the process is crucial. Educators, researchers, and academics must consider authenticity in light of their own experience but also seek to ensure that they consider personal authenticity from the student’s perspective. Currently, too few students have the opportunity to undertake IRPs, but for those that do, there are measurable benefits. Considering concerns regarding skills shortages and the lack of diversity within the scientific community, it is important to consider the significant contribution that IRPs could make, not just to science outreach, but to science education as a whole.

## Limitations

A key limitation is the lack of gender data for participants, precluding the analysis of gender-based differences. Although the study is limited in scale and based on self-reported data, the findings indicate that conducting authentic student-led research can help students develop important skills and positively influence their aspirations. However, it might be argued that by electing to participate in such a project in the first instance, students may have already had high aspirations toward science, which may have influenced the outcomes observed. Nevertheless, the findings illustrate the pedagogical value of research projects in enhancing important skills and in mediating students’ orientation toward careers in science and research.

We must acknowledge the lack of longitudinal data as to the long-term effects of participation in these projects as well as limited data on student participation in other science outreach. Further research on any long-term effect on achievement and progression into science is important given the enthusiasm and engagement of students who took part in this research project. Further work exploring the effectiveness of different models of authentic research experiences can be considered.

## Supplementary Material













## Notes

A small sample of atomic force images of the DNA origami assembled by the students (PDF)
A small sample of student posters with all names redacted (PDF)
DNA Origami Pilot Project Overview (PDF, DOCX)
DNA Origami project student participation and survey data (PDF, DOCX)
