# Supplementary material for: Science Outreach: Providing an Authentic Independent Research Opportunity in Materials Science to School Students
Source: J Chem Educ. 2026 May 18;103(6):2986–94. doi: 10.1021/acs.jchemed.5c00697 (PMC13261868; doi:10.1021/acs.jchemed.5c00697)
Supplement: Supplementary file 1 [file ed5c00697_si_001.pdf]

Supporting Information:

Science outreach: providing an authentic independent research opportunity in materials science to school students

Neil Garrido[a*], Andrew J. Lee[b], Clare Turnbull[a], Paolo Actis[b,c], Alison Rouncefield-Swales[a],

a Institute for Research in Schools, London, 165 Queen's Gate, London, SW7 5HD

b Bragg Centre for Materials Research, University of Leeds, Woodhouse Lane, Leeds, West Yorkshire, UK LS2 9JT

c School of Electronic and Electrical Engineering, University of Leeds, Woodhouse Lane, Leeds, LS2 9JT

Atomic force images of the DNA origami assembled by the students

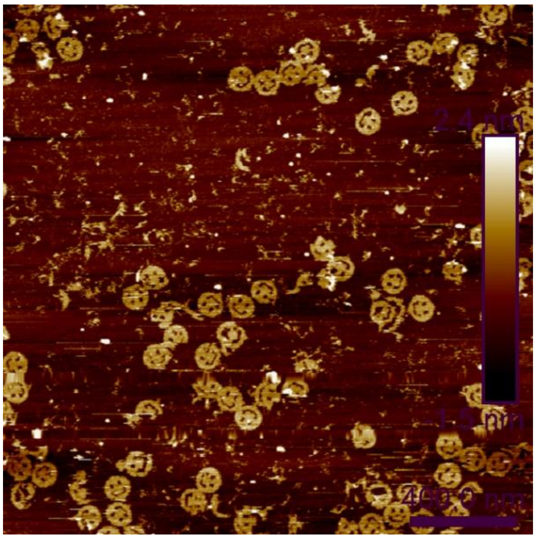

Figure 1 Atomic force image from School B

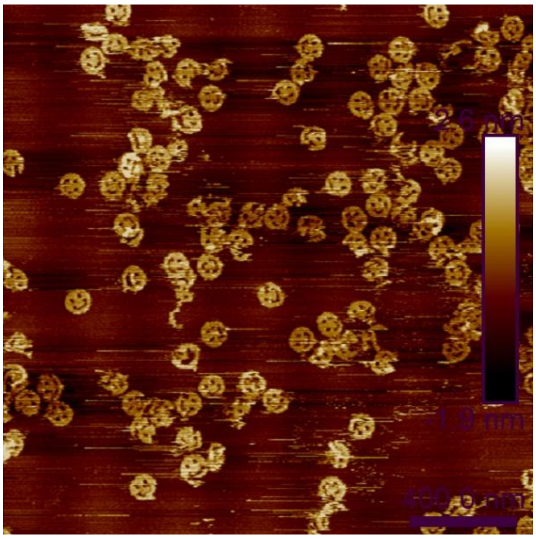

Figure 2 Atomic force image from School E

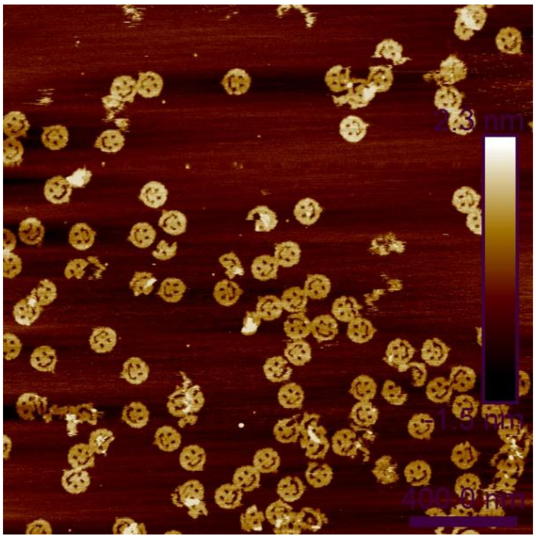

Figure 3 Atomic force image from School D

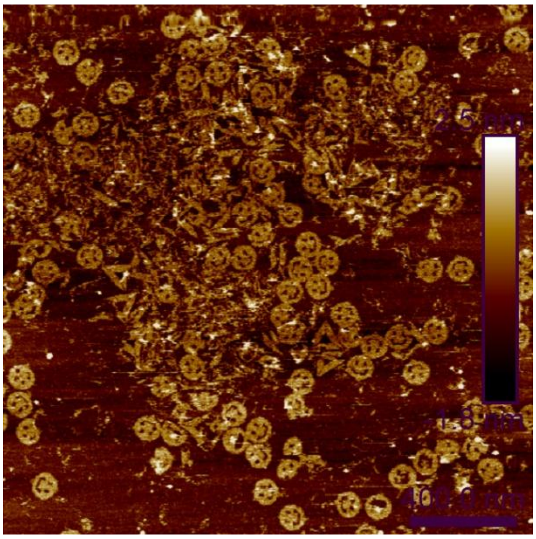

Figure 4 Atomic force image from School B
