# Supplementary material for: Science Outreach: Providing an Authentic Independent Research Opportunity in Materials Science to School Students
Source: J Chem Educ. 2026 May 18;103(6):2986–94. doi: 10.1021/acs.jchemed.5c00697 (PMC13261868; doi:10.1021/acs.jchemed.5c00697)
Supplement: Supplementary file 2 [file ed5c00697_si_002.pdf]

Supporting Information:

Science outreach: providing an authentic independent research opportunity in materials science to school students

Neil Garrido[a*], Andrew J. Lee[b], Clare Turnbull[a], Paolo Actis[b,c], Alison Rouncefield-Swales[a],

a Institute for Research in Schools, London, 165 Queen's Gate, London, SW7 5HD

b Bragg Centre for Materials Research, University of Leeds, Woodhouse Lane, Leeds, West Yorkshire, UK LS2 9JT

c School of Electronic and Electrical Engineering, University of Leeds, Woodhouse Lane, Leeds, LS2 9JT

Staples, not just for paper!

# - Analysing the use of staples and their impact on stability in DNA Origami structures

IRIS
The Institute for Research in Schools

Summary

Students at St John Fisher Catholic VA have delved into the use of computer-aided design to design and develop DNA origami structures. They used caDNAno[1] (computer software that allowed them to create virtual DNA origami) to create a simple test scaffold to evaluate the impact on stability of different staple shapes. They utilised CanDo[2] to efficiently image and compare the stability each staple pattern yielded.

What is DNA Origami?

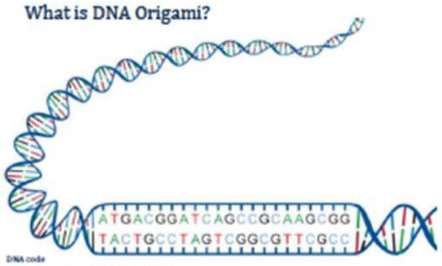

DNA code
DNA code showing specific binding of bases.

Deoxyribonucleic Acid (DNA) is formed by the binding of two single strands via hydrogen bonding of specific base pairs forming a double helix structure, Adenine (A) can only bond to Thymine (T) and Cytosine (C) can only bond to Guanine (G), these are known as complementary base pairs. Hence if we know the 'code' of bases on one strand we can identify the complementary strand on the opposite side.

With this knowledge, we then design a virtual scaffold with caDNAno, this replicates a real 7250 base pair long single strand of viral DNA (and if required, would enable scientists to identify the chemicals necessary to synthesise a design). The use of this software allows us to design virtual scaffolds rapidly and accurately.

However, the scaffold on it's own is too unstable and would coil up, which would have no practical use – DNA origami needs to be rigid and maintain its shape. To counteract this we use staples, small strands of specific DNA (oligonucleotides) which restrict movement within the structure by selectively binding to specific parts of the scaffold and forming immobile junctions.

These designs can then be synthesised via heat-annealing of the oligonucleotides and scaffold and used as materials for many purposes.

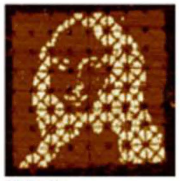

DNA Mona Lisa
The worlds smallest Mona Lisa about 1/10th the size of a human hair.

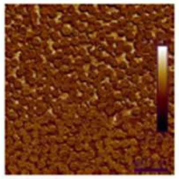

DNA Smiley Faces
DNA Origami produced by us, imaged with Atomic Force Microscopy (AFM) at the Bragg Centre. (~100nm wide each)

Background information:

Why DNA origami?

DNA origami allows scientists to create robust nanotechnologies, proving particularly useful in biosciences and computer science. The popularity of DNA origami stems from the need for a sustainable and abundant multipurpose material available at the nanomolecular scale. Potential uses include, drug delivery systems for medicine or in the development of DNA nanotechnologies as material scientists have created logic gates out of DNA origami nano-barrels.

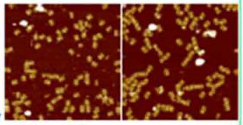

DNA Logic gates[3]
YES gate (left) and AND gate (right) being triggered via an micro-RNA input. Imaged on an atomic force microscope (2μm long)

The stability of the structure within these technologies is paramount, as it is necessary for maximal efficiency for delivery of payloads or for making structurally sound components in biological circuit boards.

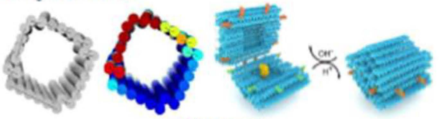

Drug delivery systems
'Nano-robots'[4] which can use ssDNA as a 'key' to unlock a payload from a DNA Origami box
DNA Nano-capsule[5] which can open and close based on environmental pH to specifically deliver drug payloads

Research aims and hypothesis

We want to know what is the most stable stapling routing possible for DNA origami nanomaterials. By changing the configuration of the staples on a simple design scaffold, using caDNAno, and then exporting our design to CanDo enabled us to evaluate and determine the structure's overall stability, and hence the preferred staple design. We can then apply our staple design to more complex structures confident that the optimum stability will be achieved.

We believe that more junctions (the crossover of our oligonucleotides connecting them together) will bring greater stability in the compound due to tighter 'knitting' within the compound. Higher junction densities are yielded by compounds with longer staples. Longer staples and more junctions also give a larger volume of hydrogen bonds (the attraction between a proton and a very electronegative atom), this will mean there is less strain within the molecule (as it is displaced less).

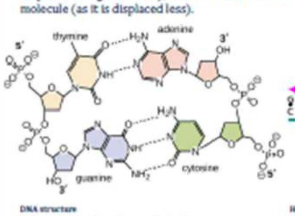

DNA structure
Hydrogen bonding shown between the four bases of DNA

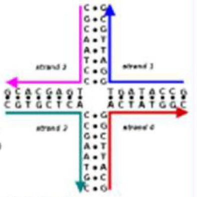

Holliday Junction
Example of 4 strands of DNA combining to make a junction

Experimental Method

First we started off with a base scaffold, this was a simple 64 base pair long square which was 6 helices tall, modelling this in caDNAno, we was then able to use this test structure to try different types of stapling methods.

We then considered what sorts of stapling methods we could use and how we would vary our staples, we decided on two key variables;

- Staple shape

The shapes of the oligonucleotides play a big factor into how secure the stapling is, these determine how many junctions are present between the staples and the routing in and between the helices.

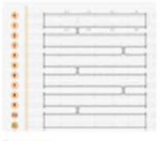

Test square
Created in caDNAno

- Staple size

We can alter the staple size in caDNAno and determine its impact on the structure and if longer or shorter staples wielded a more stable compound.

After determination of our variables we decided on 3 main shapes coming in different sizes giving a sample size of 7 different stapling techniques.

- C staples (8/16 base pairs long)
- O staples (8/16/24 base pairs long)
- S/Z staples ([8 16 8]/ [16 16 16] configurations)

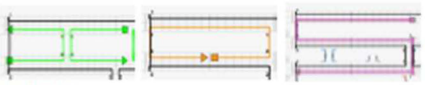

Staple examples
C, O and S/Z staples respectively

Step 2, CanDo

After designing each of our structures and creating them with the respective staple design, we then saved our designs and exported them in a .csv file format (creating the base pair sequencing for our staples and scaffolds allowing CanDo to evaluate the design). This allowed us to then to analyse the strength of the structure by exporting it to CanDo, simulating a 3D coloured image mapping weak points of the structure.

CanDo Molecular Imaging movie[6]
Scan the above on your phone to see a GIF showing the motion a DNA origami molecule undergoes.

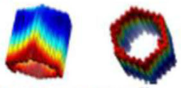

Images of a Nanorobot from CanDo[7]
These robots were used for delivering drugs around the body, and had a 'closed' and 'open' state to bind to receptors to target specific cells using logic gates.

Step 3, Analysis

Finally, we will determine how stable each structure is by analysing 3 main factors:

- Amount of weak points/overall rigidity

When colour mapped, the blue points indicate areas of high stability and hence the stapling works well in said areas. However, shades of red yellow or orange indicate areas of low stability and show the areas which are most susceptible to breaking off.

- Amount of spiralling in the structure

Due to the helical structure of DNA, the structure is bound to have some spiralling. DNA spirals approximately every 10.5 base pairs and hence for a 64 base pair long structure we should see 6 spirals (unstapled). However, the ability for a staple to counteract this will determine the lateral stability of the structure, as it will be able to resist torque forces better and will give more rigidity to the sides.

Results

Below we have images of the scaffold from caDNAno and the corresponding imaging from CanDo.

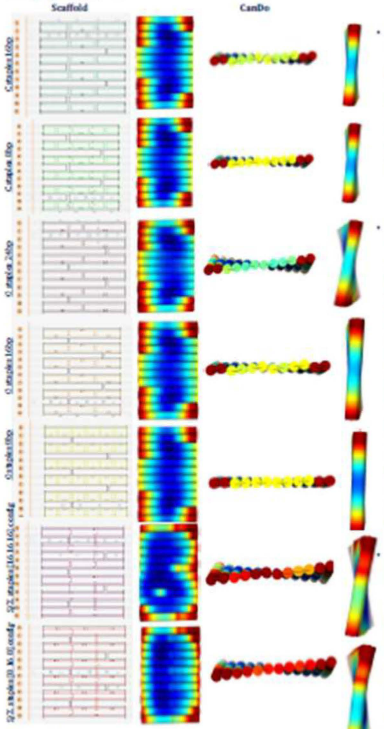

- A common trend of the C staples are a mushroom-like pattern on the inside of the structure. However the edges are stronger on the 16bp model rather than the 8bp model. And there being less spiralling on the longer figure rather than the shorter one. Overall the 16bp model is more stable.
- Interestingly, we see the longer the O staples the more spiralling is present, with the 8bp model being nearly flat and the 24bp one doing half a rotation. However, we also see that the longer the structure the stability is more spread out. The edges of each structure are similarly unstable. Although the longer structure shows stronger sides than the other two. Overall the longest O staples is the weakest, and the shortest the least spread out in terms of stability, the 16bp O staples offer a good middle ground of high overall stability and low spiralling.
- Both of the S/Z staples show large amounts of spiralling, both showing very low rigidity on the edges and sides. However they both offer good and spread out stability in the middle. The shorter configuration also shows a kink in the top half of the structure. Overall they both show poor and unpredictable stability.

Conclusions

Overall, the staples used in a structure is situational on what the structure is trying to achieve and whether lateral stability or overall rigidity is needed. Although, the staple which achieves the highest overall stability must be the 16bp O staples, as they have low spiralling and great overall rigidity. Due to the flatter structure and greater proportion of blue.

It must be noted though that the stability of a structure is dependant on a multivariate equation, and staples only play a singular part in this. However, to be able to pin down one part of it should be helpful to the quest to using DNA as a building material of the future

References:

[1] - SM Douglas, AH Marblestone, S Teerapittayanon, A Vazquez, GM Church, WM Shih, Rapid prototyping of 3D DNA-origami shapes with caDNAno, *Nucleic Acids Research*, 37 (15): 5001-6.

[2] - DN Kim, F Kilchherr, H Dietz, M Bathe. Quantitative prediction of 3D solution shape and flexibility of nucleic acid nanostructures. *Nucleic Acids Research*, 40(7):2862-2868 (2012).

[3] - Wang, Dongfang & Fu, Yanming & Yan, Juan & Zhao, Bin & Dai, Bin & Chao, Jie & Liu, Huajie & He, Dannong & Zhang, yi & Fan, Chunhai & Song, Shiping. (2014). Molecular Logic Gates on DNA Origami nanostructures for MicroRNA Diagnostics.. Analytical chemistry. 86. 10.1021/ac403661z.

[4] - Ranjbar, Reza & Hafezi-Moghadam, Mohammad. (2016). Design and construction of a DNA origami drug delivery system based on MPT64 antibody aptamer for tuberculosis treatment. Electronic physician. 8. 1857-1864. 10.19082/1857.

[5] - Ijäs H, Hakaste I, Shen B, Kostiainen MA, Linko V. Reconfigurable DNA Origami Nanocapsule for pH-Controlled Encapsulation and Display of Cargo. ACS Nano. 2019 May 28;13(5):5959-5967. doi: 10.1021/acsnano.9b01857. Epub 2019 Apr 19. PMID: 30990664; PMCID: PMC7076726.

[6] - Team Sendai (2018) *Simulation* https://biomodteamsendai2018.bitbucket.io/wiki/simulation.html

[7] - Douglas, S. M., Bachelet, I. & Church, G. M. *Science* 335, 831-834 (2012).

UNIVERSITY OF LEEDS
Bragg Centre for Materials Research

HENRY ROYCE INSTITUTE

DNA Origami: Exploring the use of DNA origami as artificial binding sites on a cellular level

The Institute for Research in Schools

Research Aims

We were interested in the concept of using DNA as a codable nano-material and therefore have researched potential uses as an artificial binding site; including treatment for anaemia and cancer. This use aims to mimic the 'induced fit' model of the specificity of an enzyme binding to an active site; we also kept in mind antibody-antigen binding, and their complementary-shaped nature.

We hypothesised that by creating artificial binding sites which could attach to working receptors on cell membranes, we could be able to permit the binding of various components, which ordinarily wouldn't bind: both essential for human metabolism; and for medical treatment of illness and disease. For example, a binding site for oxygen on a red blood cell, in the presence of faulty haemoglobin.

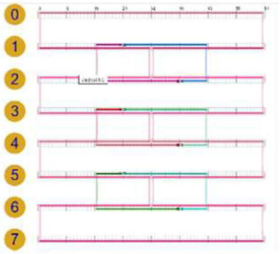

Example of a stapled strand

What is DNA Origami?

DNA is composed of two polynucleotide chains, with a sequence of four different organic bases: adenine (A), thymine (T), cytosine (C), and guanine (G). These bases align via complementary based pairing (A and T, C and G) and form hydrogen bonds between them, allowing the structure to coil into a double helix, thus stabilising the structure.

Using this knowledge, we are able to predict a complementary strand of base sequences using the known DNA code of one strand as a template.

DNA origami is an assembly technique that folds single-stranded DNA template molecules into target structures. This is done by using scaffolding templates to stabilise hundreds of short 'staple' DNA strands.

SCADNANO

To begin our project, we first started experimenting with different shapes on scadnano to practise creating the scaffolding strands, which can then be coloured and altered to produce different shapes and designs. We experimented with different designs.

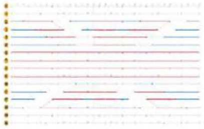

A Union Jack Design using different scaffold colouring

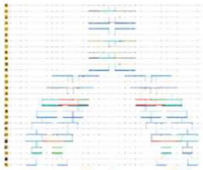

An early design of an antibody to model our concept

Staples

We focused several weeks on learning how to correctly stabilise scaffolds using staples. This is an essential for a structure to be able to fold into a functioning 3D nano-material.

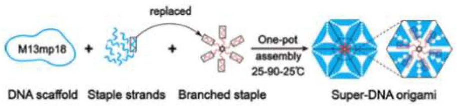

Evaluating Base Sequences

After we had created a stapled design we were then able to export this to WordPad to view the base sequence. This is beneficial as it allows use to relate the shapes we created to the organic DNA bases used to create it. To create the binding sites, we would need to ensure the bases are complementary to the membrane it would have to bind to.

Start,End,Sequence,Length,Color
8[31],9[47],GGTTGAGTGTTGTTCCAGTTTGGAACAAGAGTCCACTATTAAAGAACGTGGACTCCAACGTCAAAGGGCGAAAAACCGTC,80,#ff0000
1[32],3[47],TCCACGCTCGCCAGGGGTATTGGGGGTTTGCCCCAGCAGGCGAAAATC,48,#ff0000
0[47],1[31],TGGTTTTTCTTTTCACCAGTGAGACGGGCAACAGCTGATTGCCCTTCACCGCCTGGCCCTGAGAGAGTTGCAGCAAGCGG,80,#ff0000
6[47],8[32],CCCTTATAAATCAAAAGAATAGCCCGATGGCCTATCAGGGCGAGATAG,48,#ff0000
4[55],5[55],TGGTGGTTCCGAAATC,16,#ff0000
6[55],7[71],GGCAAAATCACTACGTGAACCATCACCCAAAT,32,#ff0000
2[71],3[55],AACGCGCGGGGAGAGGCGGTTTGCCTGTTTGA,32,#ff0000
3[72],2[72],TTCCACACAATCGGCC,16,#ff0000
5[72],4[72],TGTTATCCGCTCACAA,16,#ff0000
7[72],6[72],CAAGTTTTTGTGAAAT,16,#ff0000
3[80],1[95],AACATACGAGCCGGAAGCATAAAGCATTAATGGCCAGCTGTGTAAAGC,48,#ff0000
9[80],8[96],GTAAAGCACTAAATCGGAACCCTAAAGGGAGCCCCCGATTTAGAGCTTGACGGGGAAAGCCGGCGAACGTGGCGAGAAAG,80,#ff0000
8[95],6[80],CAAGGCAACAGCTGCCTTGGGGTCAATCATGGTCATAGCTGTTTCCTG,48,#ff0000
1[96],0[80],CTGGGGTGCCTAATGAGTGAGCTAACTCACATTAATTGCGTTGCGCTCACTGCCCGCTTTCCAGTCGGGAAACCTGTCGT,80,#ff0000

Applications of DNA Origami as Binding Sites

We researched some common binding sites which may malfunction or mutate to a non-functioning state. Using this knowledge, we can consider which binding sites could be replicated using DNA origami.

Anaemia is a condition which can affect a person who, either doesn't have enough red blood cells; or has too few, or non-functioning haemoglobin. Haemoglobin is a globular protein which consists of four polypeptide chains, each containing a ferrous (Fe2+) haem group. This haem group allows oxygen in the blood to associate so it can be transported in the blood and then delivered around the body for use in cellular processes, such as respiration.

Whilst most types of anaemia aren't life threatening, the use of an artificial binding site for oxygen would relieve some symptoms: tiredness, heart problems, headaches, and lack of energy.

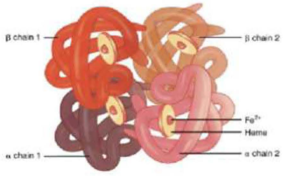

Cancer is a condition where abnormal cells begin to divide uncontrollably, causing damage to healthy cells. Cancer is the second leading cause of death globally due the difficulty involved in treatment due to the complexity of some types of cancer.

We have theorised that it may be possible to synthesise an artificial binding site which could attach to the cell membrane of cancer cells to act as an inhibitor for growth (suppressor protein), which cancer cells lack. This would decrease the rate at which cancer cells grow and divide, possibly even stopping it completely.

Alternatively, it may be possible to create a 'virus style' delivery system from DNA origami, where 'healthy' DNA could be 'injected' into cancerous cells by some delivery mechanism.

This process would use DNA origami to synthesise a 'delivery capsule' which would contain an extracted 'healthy' DNA strand from the patient. This could then be injected (like viral DNA to a host) into the nucleus of the cell upon arrival, allowing the cell to undergo DNA replication to produce new copies of the healthy DNA, allowing more cells to contain this DNA.

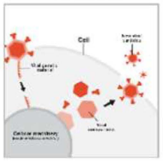

Courtesy: National Human Genome Research Institute

Conclusion

In practise, these designs may not function as we imagined due to the difference in length of DNA strands and quaternary proteins. In reality, it is rare for foreign DNA to reach its target due to how enzymes readily break it up. Furthermore, the production may be too time consuming and costly to be worth the benefits for some uses, such as anaemia. Conversely, for illnesses such as cancer, the research may be vital due to the difficulty in treatment.

We haven't created specific complementary DNA origami yet as this would require more in-depth research into precise membrane attachment proteins of specific cells, however we do believe it would be possible, coupled with more comprehensive research into it with electron microscopy images of cell membranes.

Next Steps

To begin the synthesis of a prototype binding site we would begin to gather base sequence data from cells and then translate this onto the software to begin its 3D design. This is beneficial as it can reliably predict which designs are stable.

In our research phase we found the software 'ChimeraX' which allows you to view DNA origami in 3D, a challenge of the 2D format of scadnano. We tried to use this program to view our stapled designs, however we had difficulty with file types so were unable to utilise this fully, but work is ongoing to resolve this. We would aim to overcome this is in the future by doing more research into the compatibility of the software, so we could visualise our findings more easily.

References:

Rukkumani Rajagopalan, Jatinder V. Yakhmi. (2017) *Nanostructures for Cancer Therapy*

J. Am. Chem. Soc., (2024) *Chemically Conjugated Branched Staples for Super-DNA Origami*

UCSF Douglas Lab, [online] (2020) *Scadnano*

the BRAGG centre FOR MATERIALS RESEARCH

HENRY ROYCE INSTITUTE

Designing a Tesla Valve using DNA origami -using CaDNAno software to create useful components for a Nano-device.

IRIS
The Institute for Research in Schools

Summary

This project allowed us to use experimental technology and computer programs that we had no experience with, and it taught us about DNA origami. It allowed us to combine fields of Computing and Biology and gain experience with materials science, an interdisciplinary field which creates materials based on specific properties. **DNA origami is a new development in nanotechnology that allows us to fold long strands of DNA into complex structures.** This has uses in many different fields; such as drug delivery and self-building materials. **We aimed to make a Tesla valve (a valve with no moving parts)**, with the idea that this valve could be used as part of a nanochannel for unidirectional molecule transport the hopes that it would have many **biomedical or analytical chemistry applications.**
Using **CaDNAno** software we designed the DNA structure and then with **CanDo** we were able to predict structural integrity and improve the design.

Folding DNA into a Smiley Face

We also had the opportunity to learn about the synthesis process involved in DNA origami, through work with facilities at the Bragg Centre for Materials Research. They enabled us to create our own DNA sample using a simple design, a smiley face. Once synthesised, it was observed through atomic force microscopy so we could see what we had created. This opportunity led us to develop new skills in the practical and biological aspects of this project, particularly with pipetting at smaller scales than regular A-Level sciences, and to work with the larger scientific community at the Bragg Centre. This practical synthesis helped us to understand the requirements to bring our own Tesla valve design to life in the future.

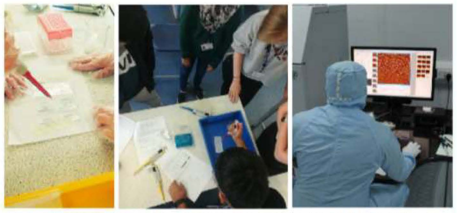

Above the images show our group working to accurately pipette 1/1000 cm^3 volumes of the 237 staples and buffer chemicals necessary to synthesise the DNA origami smiley faces. The mixture was then cooled and separated in a purification column *via* centrifugation, and the final sample was sent off to the Bragg Centre to be analysed and visualised.

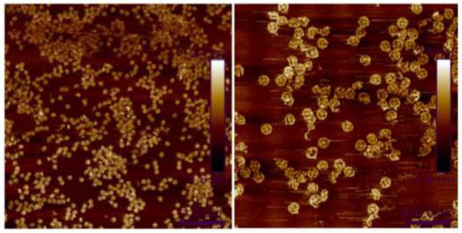

Atomic force microscopy image of DNA folded to form smiley faces. The nano-structures were successfully synthesised and imaged by microscopy imaging technology we had not previously encountered in school.

Research aims

In our research we aimed to make a Tesla valve which is a fixed-geometry passive check valve. It allows a fluid to flow preferentially in one direction, without moving parts. We used DNA Origami which is the nanoscale folding of DNA, to try and make a 3D Tesla Valve.
This valve could be implemented as part of a nanomachine to regulate unidirectional transport of molecules – redirecting any flow to a single destination and preventing backwards travel. As it would be made up of DNA, it could be less likely rejected from the body than other artificial valves. Potential use in plants and animals for active transport so the nutrients can only flow one way may reduce the need for ATP, there could also be potential applications in HPLC for small biological molecules. The advantages of self assembly and no moving parts should facilitate ease of production as well as stability.

Background information

Given our aim to create useful mechanical components on a nano scale that could later be incorporated into larger nano-projects paired, with the limitations of the current DNA Origami technology which prevent objects with moving parts from being easily constructed, the Tesla valve seems like the most logical and potentially useful project aim.

A Tesla valve, invented by and named after Serbian-American inventor Nikola Tesla in 1920, is a unique example of a one-way water valve free from any moving parts. It functions by providing a passage to fluid in one direction with virtually no resistance except friction yet using loops or recessions to cause huge resistance to fluid in the other direction slowing it down or redirecting it into the desired direction.

The lack of moving parts, as well as making it more attainable with the CaDNAno software, makes the Tesla valve more durable as well as being easy to scale and manufacture from a variety of material:

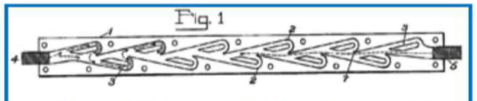

Original illustration by Nikola Tesla in his patent, showing the arrangement of the tube to allow for low resistance to flow from left to right and high resistance restricted flow from right to left

Experimental Method

Two programs are used to design and visualise the DNA structures. CaDNAno allows us to simplify the structure to a 2D interface. A long, continuous 'scaffold' strand of DNA is pinned by smaller 'staples' to connect and form the ultimate 3D shape. The program has two choices of base lattice – typically square for 2D, and honeycomb for 3D. Our structure deviates from this – instead, using the square lattice to form a 'net' and pinning this so it folded to create the desired shape of the valve. The colour coordination of 'scaffolds' and 'staples' allows us to distinguish between them.

CanDo visualises these structures and their properties without having to synthesize and test it – costing less time and resources to iterate the design as flaws are discovered. By predicting the 3D structure of our programmed CaDNAno design, it can give us the shape, flexibility, fluctuations and mechanical properties of our model. The rapid feedback on feasibility helps to gain insight to the designs stability at a rate much faster than synthesizing and analysis of a sample.

Results and Analysis

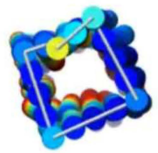

After completing the exercises to design two dimensional objects the challenge was to move into three dimensions.
An example of our early iterations with the CanDo software was testing the structural stability of a section of tubing created on CaDNAno shown here on the left. We decided to make a tube as an early attempt into 3D design. The Tesla valve is based on a modified section of tubing, we knew this could be both a useful test of software, a training exercise and potentially a base to model the Telsa valves design.

Tesla Valve Design Process

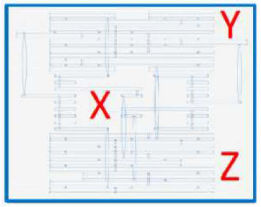

STEP 1: The Scaffold
The DNA scaffold was designed in CaDNAno. This two-dimensional image shows what will fold to form the three layers – the middle section (X) containing the valve components, and top (Y) and bottom (Z) enclosing the structure.
This design method is similar to drawing the net of a shape, from the long, single scaffold, and the staples act to ensure each fold is made correctly.

STEP2: Modify the Code
Early in the development of the Tesla valve structure, we found that the auto staple function would immediately crash the program. While this feature would be successful with smaller structures, an error would occur on larger structures, usually those with three dimensions resulting in a loss of functionality and data from the software.

```
vhi5 = self._node5.virtualHelixItem()
vhi3 = self._node3.virtualHelixItem()
sameStrand = (self._node5.strandType() == self._node3.strandType()) and vhi3 == vhi5
if sameStrand:
    pen.setStyle(Qt.DashLine)
    pen.setDashPattern([3, 2])
```

```
try:
    vhi5 = self._node5.virtualHelixItem()
    vhi3 = self._node3.virtualHelixItem()
    sameStrand = (self._node5.strandType() == self._node3.strandType()) and vhi3 == vhi5
    if sameStrand:
        pen.setStyle(Qt.DashLine)
        pen.setDashPattern([3, 2])
except:
    print('Code is fixed, this would normally crash the program')
```

After spending multiple hours stapling sections of this project and several other projects by hand, we realized that the error given on the python shell was pointing to the same line of code each time. This section of code appeared to be for generating the class for displaying the lines within the python window and determining the colour of these lines. Because of that, preventing this sections from running occasionally should not have had any negative effect on the structure of the Tesla valve itself. In addition to this, the line only appeared to give an error when the section it was trying to generate did not physically exist. After adding a try statement around this line of code and the lines around it the crashing issues was fixed.

As this was a simple fix and did not seem to change anything significant within the structure of the program, it was unclear as to why this issue was present within the version of CaDNAno that we were using.

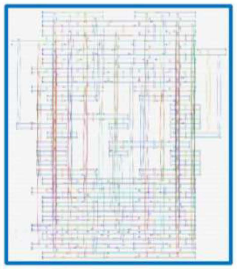

STEP 3:Addition of Staples
DNA staples were added from the corrected code to provide structural support. These ensure the valve is held in place and retains its 3-Dimensional shape, enabling a substance to be carried through the valve without it collapsing.

After using the auto staple feature and the auto break feature to split the staples into reasonable sections, several different iterations were sent to the CanDo website for analysis. Once each of the iterations were returned, we used the data from the videos to determine where the points that would provide structural instability would be and the appropriate fixes were made at every point.

STEP 4: Stabilisation and Optimisation
Below the CanDo renderings of a segment of Tesla valve are shown with the top and side elevations. The opening to the valve indicated by the arrow. Heat map colour coding showing the relative structural stability in different areas of the design. We used these to identify fragile points and re-iterate our design to improve upon them, ensuring the long-term durability of the Tesla valve, which made this a thoroughly engaging project.

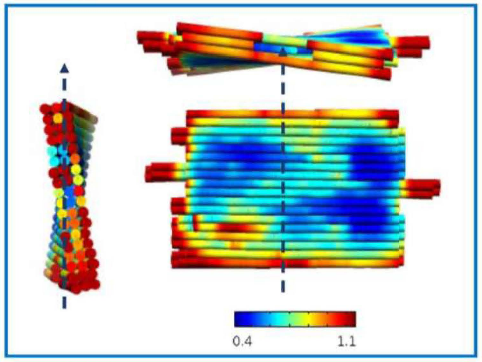

Conclusion

We believe that the Tesla valve made from biological material like DNA could be very helpful within medicinal fields and we managed to model the assembly of a "subunit", there is much work needed to achieve the end goal, such as discussions of the fluid dynamics that allow the Tesla valve to facilitate unidirectional flow at this nano-scale as well as interactions between the DNA structure and the contents of the tube. This is also only a section of the valve and a system for assembling these subunits needs to be designed. It is hoped that this design, may however, stimulate discussion about an application of DNA origami that has not been previously considered!

We started this project with the aim to learn about some novel science and to use new technology. We gained experience in DNA manipulation and created smiley faces. While we were modelling the Tesla valve we experienced some code difficulties, and really enjoyed problem solving with. The whole process has been incredibly valuable, opening our eyes to a new field of science and how our diverse range of subjects at A-level came together like that of a multidisciplinary science team.

UNIVERSITY OF LEEDS
Bragg Centre for Materials Research

HENRY ROYCE INSTITUTE

DNA Origami
-using FRET microscopy techniques to detect cellular forces

IRIS The Institute for Research in Schools

Summary

This project allowed us to use experimental technology and computer programs that we had prior experience with, and it taught us about DNA origami. It allowed us to combine fields of Computing and Biology and gain experience with materials science, an interdisciplinary field which creates materials based on specific properties. **DNA origami is a new development in nanotechnology that allows us to fold long strands of DNA into complex structures.** The biocompatibility of DNA origami technology alongside its easy programmability leads to many applications in biomedical and cellular research as well as bioengineering. We aimed to make tubular DNA structures of varying stiffness to act as a surface for cells to interact with. This idea could be combined with fluorescent markers and Forster Resonance Energy Transfer (FRET) to detect small distance variations and thus forces to very high resolution.

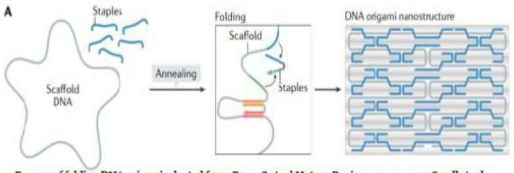

Process of folding DNA origami adapted from *Drey. S et. al Nature Reviews. 2021. 1: 13.* Small staple structures are inserted into scaffold DNA where upon annealing induce folding into the desired DNA origami nanostructure.

Folding DNA in the lab

Throughout this project we have had the opportunity to learn about and conduct the DNA origami synthesis process, in conjunction with the Bragg centre for materials research. We have carried out the synthesis process for a simpler DNA origami design, a smiley face, in our labs in school. We have had the opportunity to observe similar samples being imaged through Atomic Force Microscopy at the Bragg centre in Leeds. This opportunity led us to develop new skills in the practical and biological aspects of the project, primarily with the pipetting of small volumes that are not regularly encountered in regular practical A-level sciences. This synthesis has helped us to understand some of the requirements to bring our DNA origami designs to life in future.

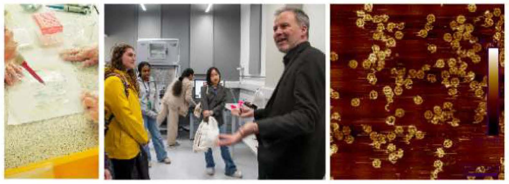

The above images show our group working to accurately pipette 1/1000cm³ volume samples of all 384 staples and buffer chemicals to produce the DNA structures which was cooled and separated through a purification column in a centrifuge. The central picture shows our groups visit to the Bragg centre facilities to learn more about the Atomic Force Microscopy technique used to view the samples and the final image is an example of the smiley face structures produced by previous project students.

Research aims

In our research we aimed to design a DNA structure that could act as a pressure sensor. These would allow for the detection of small displacements in z-axis which, when combined with the material properties of the DNA structures could be used to look at forces that cells apply to the surface. By tailoring different tube-like DNA structures we could create a variety of different sensors with a range of sensitivities .

Using Forster Resonance Energy Transfer (FRET) microscopy with fluorescent markers attached to the DNA structures could allow for very high resolution detection of tube displacements. With different markers attached at opposite sides of the tube structures emission of donor and acceptor fluorophores could be used to gauge the deformation of the tube under pressure. Once created these structures would be able to undergo calibration whereby AFM can be used to apply known forces and FRET microscopy used to measure signal ratios (corresponding to deformation).

Applications of this design could be used in cell research to detect proliferation of cells across surfaces, or to measure the impact of forces applied by cells in biological environments. In theory this would help detect the stiffness and pressure on a cellular level which could be used in medical research such as cancer research to test the stiffness of tumours and healthy cells.

Background information

DNA origami is a branch within nanotechnology that allows us to fold and shape DNA using staples to form complex and useful structures. These structures can include those that help in drug delivery or the creation of a synthetic valve. DNA can achieve extreme precision in which other materials may lack in, which allows us to create well-defined nanostructures of near-atomic precision.

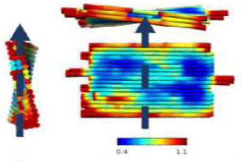

CanDo model of synthetic valve *Image adapted from Blanks. N et al. Tapton School. 2021*

In our research we looked at methods already used for the detection of cellular forces. Some techniques have been used to look at the contractile forces that come from cells on surfaces however these work on the x and y axis, such as the polymer pillar example shown. Our idea to create a tension sensor in the z axis was one that would require us to think of a design that would allow deformation of the structure in this direction as well as a method through which to accurately measure this deformation.

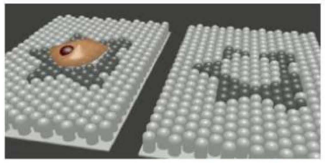

Deformation of polymer pillars to measure contractile forces in cells. Where polymers show the greatest deflection the greater the force being applied by cells as they spread and change their environment. *Adapted from B.Robinson CMBL Imperial college.*

Forster Resonance Energy Transfer (FRET)

FRET is the process by which energy from an excited fluorophore (known as a donor) is transferred to a chromophore (known as an acceptor) that is in close proximity. The fluorophores are chosen so that the emission wavelength of the donor overlaps with the absorption wavelengths of the acceptor. In FRET microscopy lasers are used to excite the acceptor and detection of emission is conducted at wavelengths corresponding to the emission of the acceptor and the donor. The amount of energy transferred (FRET efficiency) is known as the fraction of photons absorbed by donors whose energy is transferred to acceptors, and this value is greatly dependent on the distance between the fluorophore and the chromophore. Due to this, FRET works on nanometer length scales where the donor-acceptor distance is less than 10nm, this makes it a desirable technique for detecting very small deformations and thus high resolution force detection when combined with our technique

Along with the increased availability of fluorescence microscopy instrumentation, FRET has been able to become a regular technology in modern molecular biology, particularly in the study of cellular signaling (which is the ability of a cell to receive, process, and transmit signals with its environment and with itself). For example, the onset and end of Ca^{2+} signaling in specific cellular structures such as the cytoplasm, nucleus, or endoplasmic reticulum can be worked out by measuring the change in the ratio of the fluorescence intensities of acceptor and donor molecules in live cells.

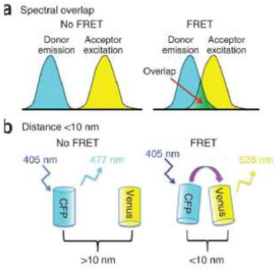

For FRET to occur. a) The emission spectrum from the donor molecule must overlap with the excitation spectrum of the acceptor molecule. b) If the FRET donor and acceptor are more than 10 nm apart, then no FRET occurs. *Adapted from Broussard et al. (2013)*

Experimental Method

We started to developed our design using scaDNAno, which both helped us to develop skills in experimental programming and computer programs in which we had little experience with. It also gave us the chance to explore the ways in which the fields of computer science and biology are able to combine to create fascinating structures based on the simple properties of a strand of DNA.

We conducted initial modelling of our desired structures on fusion 360 and on Autodesk fusion to guage how our structures may look as we vary the number of DNA strands in our tube structures. These are to be translated in the scaDNAno software which is a process that is currently in active development.

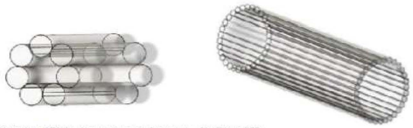

Examples of DNA tube structure designs created in fusion 360

Results

The project is still in active development however, as the seen in the diagram below, we have started to create designs for the tube structures which will accommodate affixing of flurophores to each pole (indicated by the blue and yellow below). These will be able to measure the applied force on the z axis through FRET where exchange of energy between an excited fluorophore (the donor(blue)) to a nearby chromophore (the acceptor(yellow)) works through a non-radiative dipole-dipole coupling. The tension sensor works by the energy transfer between the donor and the acceptor and will be tailored to different sensitivities through stiffnesses and sizes of tube structure that we are in the programming stage of at present.

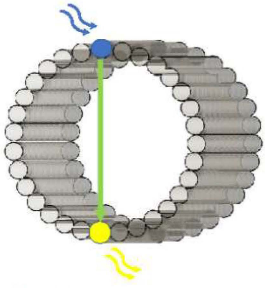

Design of DNA tube structures with donor and acceptor placements shown. Green arrow indicates non-radiative transfer between fluorophores.

Analysis & next steps

This project is ongoing and so Our next steps will be to complete the programming in CADNANO and model the different structures we create to assess their rigidity and stability – factors that would influence their force sensing capability. We will attempt to make and program a wide variety of these tube structures as the amount of staples you put in effects there overall properties, especially stiffness, by doing this we will assess the optimum design.

Furthermore, we need to consider that acceptor fluorophores could display different brightness levels when imaged together. This means that when dual fluorophore imaging takes place, one of the acceptors may be saturated, generating a level of brightness which is misleading. Moreover, measurements can be misleading due to the crossover and cross-excitation of overlapping fluorophores. For example, fluorescence from the donor can leak into the detection channel of the fluorescence of the acceptor. As this will most likely happen for any acceptor donor pair, it must be considered when measuring FRET. If this were to be simulated in a lab, we would need to consider all potential errors in the method.

M053

The Institute for Research in Schools | UNIVERSITY OF LEEDS Bragg Centre for Materials Research | HENRY ROYCE INSTITUTE

DNA Origami

# - Aortic Heart valve replacements

IRIS The Institute for Research in Schools

Summary

DNA Origami is when DNA is extracted from a virus and it is then folded to create a shape: the scaffold. The scaffold will be a loop of single stranded DNA and so won't hold the shape so short sequences of DNA bases are bonded to parts of the scaffold to make it double stranded which will allow it to remain in the shape that we want, these short sequences are the 'staples'.

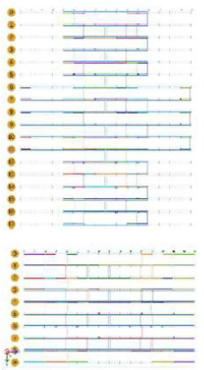

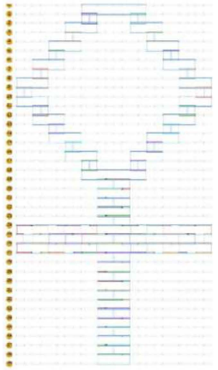

When we were first becoming familiar with using the Scadnano software we tried different, more simple designs (left) but as we became more confident with using it we did some more complex shapes (right) which was challenging as we had to really think about how we could apply the staples. Due to DNA being non-antigenic we realised there would be a smaller chance of it being rejected like a biological valve replacement.

Aortic Valve Background Information (4)

People may need an aortic heart valve replacement if:

- They have a congenital heart defect (bicuspid – symptoms of heart failure).
- Aortic regurgitation.
- Valve damage (endocarditis)

Current options include:

- **Mechanical** – risk of thrombosis. Anticoagulants needed which come with a risk of bleeding.

Aortic Valve Background Information (continued)

- **Biological (pig or cow)** - risk of thrombosis. Must take immunosuppressants for the rest of their life. Could tear over time. Not suitable for all religions.

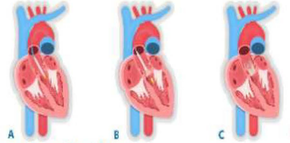

(Source 1)

Operation Options:

- **Open heart surgery:**

- Can be used for people with anatomy defects
- More painful recovery (6-8 weeks)
- More doctors can perform it
- Chest pain, stroke, heart attack, lung/kidney failure, memory loss, wound infection

- **Transcatheter aortic valve implantation (TAVI):**

An incision is made in the groin or collar bone and a catheter is passed into the aorta. The catheter is then guided into the opening in the aortic valve and a new valve, made of metal and animal tissue, is positioned inside the old valve.

- Minimally invasive
- Less painful recovery (7-10 days)
- Lower mortality rate
- Infection, stroke, kidney disease, heart attack

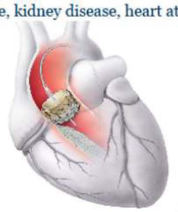

TAVI valve

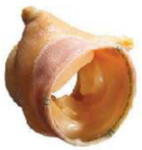

Biological pig valve (Source 3)

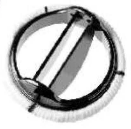

Mechanical valve (Source 2)

Our Initial Idea

We need our valve design to have a rigid line down the middle so that when it is attached at either side the two flaps can open when blood leaves the ventricle and close so there is no regurgitation.

If we were to take it further we would also either design a ring which would act as the outside of our valve which will be the part that is in contact with the aorta, or we would do some research into using other materials to act as the outside of our valve.

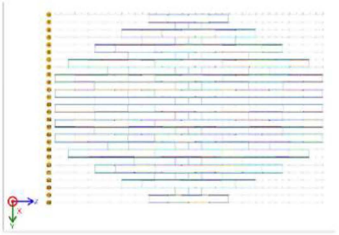

Initially we just designed a disc with regular staples to be the centre of our valve but we realised what this would not work as well as we wanted it to because the structure would twist due to the nature of DNA, so we experimented with different staple patterns and tried out using deletions for the parts that we wanted to be more rigid and insertions for the more flexible sections.

Experimental Method

We realised that due to the nature of DNA any designs we made would have a twist in them and, even if it was only slight, that would cause a problem if it were to be used as a heart valve. To try and counteract this we collaborated with the other group from our school who had researched and experimented with using insertions and deletions. They found that the use of deletions made that part of the structure stronger and insertions would make it more flexible. So, we decided to test this out using a rectangle as proof of concept, as shown in **Results**.

Results

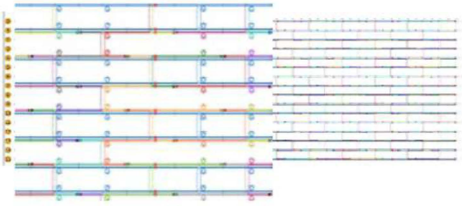

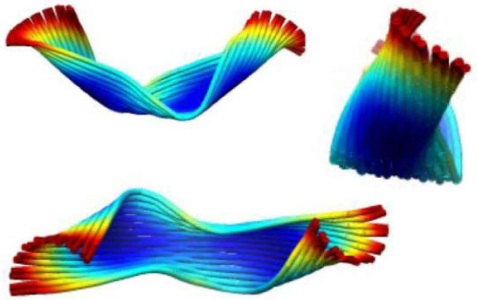

Analysis & conclusions

This was not an ideal result to get but, as you can see above, the middle of the structure folds which is exactly what we want to achieve. However the middle is also rigid (shown by the dark blue) which is not ideal as we need it to be flexible so that blood can flow through it, so if we were to do further experimentation on this idea we would try out spacing the deletions differently. We would also try and find a way to avoid the bend that is in the middle as something like this would not work as a heart valve.

To make the use of DNA origami feasible in Aortic valve replacements some experimentation would have to be done to see if we could make the valve strong enough to withstand the pressure of the blood that is leaving the heart (120 mmHg) because a long-lasting valve is in the best interests of the patient and surgeon.

References:

1. https://www.bhf.org.uk/informationsupport/treatments/tavi
2. https://www.pennmedicine.org/for-patients-and-visitors/patient-information/conditions-treated-a-to-z/aortic-regurgitation#:~:text=What%20Is%20Aortic%20Regurgitation%3F,blood%20flow%20through%20the%20valve.
3. https://www.google.com/url?sa=i&url=https%3A%2F%2Fwww.heart-valve-surgery.com%2Fheart-surgery-blog%2F2012%2F03%2F26%2Fpig-valve-replacement-harold-roberts-juergen-ennker%2F&psig=AOvVaw3fGg4IfBMzZ-2Yw60QCklj&ust=1684603592126000&source=images&cd=vfe&ved=0CBIQjhxqFwoTCODvx8fzgf8CFQAAAAAdAAAAABAE
4. https://www.nhs.uk/conditions/aortic-valve-replacement/

M030

Institute for Research In Schools

UNIVERSITY OF LEEDS Bragg Centre for Materials Research

HENRY ROYCE INSTITUTE

How DNA Origami May Impact Neuroscientific Medicine

The Institute for Research in Schools

Summary

DNA origami is the method of **folding DNA into nanostructures**, allowing it to be functionalised and modified, and has emerged as a promising avenue in fields of neuroscientific medicine due to its **programmability** and **flexibility**. DNA exercises structural **predictability and biocompatibility** that enables the manipulation of structures for use in therapeutic applications. In terms of neuroscience, it poses a solution to delicate issues within **neurological diseases** such as epilepsy and Alzheimer's by modulating ion channel activity and reinforcing the **blood brain barrier** (BBB) to prevent damage or inflammation. The ability of DNA to interact with the BBB without impairment offers potential for treatment of Alzheimer's by inhibiting neuronal apoptosis and supporting nerve regeneration. However, it is important to consider the **social and ethical implications** of using DNA nanostructures in medicine. Overall, DNA origami could revolutionise neuroscientific medicine by offering customisable and versatile **construction of tailored nanostructures** which are able to address complex neurological conditions.

Research Aims

We investigated the use of DNA origami in various neurological disorders, aiming to make a conclusion on the various proposed strategies and summarise potential explorations. We then looked into the social and ethical aspects of using DNA origami in medicine and took a survey on current knowledge of DNA in medicine, aiming to research public views of DNA nanostructure manipulation for therapeutic use.

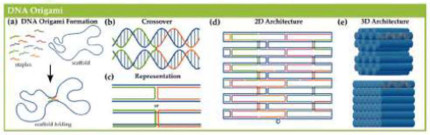

DNA Origami Synthesis
How initial scaffolds combine with staple strands to form structures that fold into organised DNA origami.

What is DNA Origami?

DNA is useful for **storing information** because of the way it can be woven into structures. It is **cheap and easy** to use, as it **reacts predictably**, and can be modified and functionalised. DNA, as **a double helix**, is very rigid and stable, providing an avenue for the creation of nanostructures. DNA origami is the creation of these nanostructures through the folding of 2D shapes (1).

In DNA origami, the negative phosphate backbone sits on the outside, and the base pairs on the inside. An original **scaffold strand** is created, before being reinforced with staples. Scaffolds are taken from **viruses** such as the **M13 bacteriophage**, as they have single-stranded DNA meaning the DNA for the scaffold does not need to formed and purified.

Staples (complementary strands of single-stranded DNA made from oligonucleotides) are used **to bind** specific parts of the scaffold; these create **immobile junctions** which prevent it from changing shape. The staples and scaffold are **mixed, heated** to denature them, and then cooled slowly. The DNA origami **self-assembles**, and can be joined to make nanostructures.

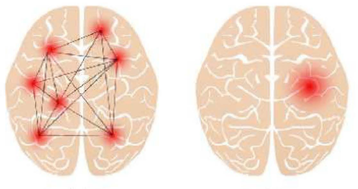

Mechanisms of Epilepsy

Epilepsy affects over **10 million people** worldwide. It involves recurrent seizures, and whilst medication to subdue seizures is often used as a treatment, it is not always effective. Therefore, targeting the underlying **mechanisms** that control the seizures would be better.

Seizures are a sudden reoccurrence of symptoms that alternate with free periods due to a lot of neurons in the brain firing at the same time. There are 3 different categories of seizures focal, generalized and unknown. Focal seizures start limited to 1 part of the cerebral hemisphere. Generalized seizures occur when both halves of the cerebral hemisphere have activity at the same time. **Epileptic seizures** are the result of an imbalance between **excitatory and inhibitory** signals (3) in the network of cortical neurons. The brain traditionally maintains a delicate balance of these signals:

- Excitatory signals mediated by the **neurotransmitter glutamate**, which causes the firing of neurons
- Inhibitory signals mediated by the **neurotransmitter gamma-aminobutyric acid (GABA) which suppresses neuronal firing**

Excitability is governed by **action potential**.

- There is an **electrochemical gradient** (- to + potential) across the membrane, which is caused by ions (sodium and potassium mainly).
- **The resting state** of the membrane is overall negative (-60 to -70 mV) due to the passive movement of potassium ions.
- **A stimulation** in the neuron occurs and the voltage sensitive **sodium channels open** causing a **depolarization** (+ potential).
- The potential returns to rest by **repolarization**. Meaning the voltage sensitive sodium channels close while potassium channels open to **balance** the potential.
- **Hyperpolarization** occurs due to the potassium channels staying open so that the neurons don't fire again.

This is what an action potential is, where there is a change in voltage across a membrane. Action potentials **spread** down the axon to start the neural transmitters, in some cases it creates an **imbalance** in the **inhibitory** and **excitable** synapses causing the final **epileptic crisis**.

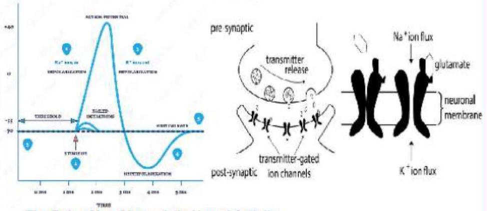

The Role of Ion Channels in Neural Activity
Abnormal ion movement affects neurotransmitter detection and release, therefore inducing seizures as a result of excitability and inhibition.

Membrane Manipulation

- DNA origami structures could be used to **interact** with specific ion channels, modulating their activity to restore balance
- **Bind** to voltage-gated sodium channels and **stabilis** activity by **blocking overactive** channels, allowing time for **repolarization**
- Molecules could be added that block/enhance channel activity, and **controlled release mechanisms** would prevent hyperexcitability
- **Reconfigurable** DNA structures could change conformation in response to neuronal activity. Drugs can be placed inside, and the structures **locked** with particles of **iron oxide**.
- **Microscopic DNA robots** could treat epilepsy. Structures were detected to unlock in cockroaches in response to **specific brain activity**, and rate of the release was tracked (5)
- The release process could be **controlled by human brainwaves**, in response to hyperexcitable regions.
- In theory this could allow the **switching of bioactive molecules** on and off in response to **cognitive states**, enabling therapeutic control in disorders that are the most difficult to treat

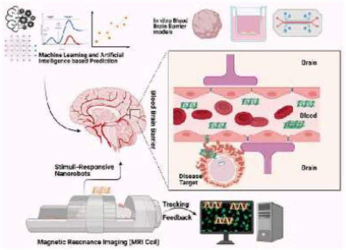

The Blood Brain Barrier & Alzheimer's

The Blood Brain Barrier (BBB) is a **protective shield** for the central nervous system (CNS) separating it from **blood circulation**, and therefore preventing harmful substances and immune cells from entering the brain. It **maintains the ideal environment for nerve cells (6)**. **Epileptic seizures disrupt the BBB**, and many other neurological diseases such as Alzheimer's are associated with damage to the BBB.

Effects of Epilepsy:

- Epileptic seizures disrupt the BBB by increasing its **permeability**, leading to **protein and immune cell leakage**.
- **Blood albumin** and immune cells can enter the brain, potentially leading to inflammation.
- Albumin **triggers astrocytes**, which cause further inflammation and reduce potassium and glutamate regulation, increasing **neuronal excitability.**
- Inflammation reduces the **threshold for seizures**, and causes abnormal neuronal **depolarization** effects
- **A positive feedback loop** is created, where seizures which damage the BBB make more seizures more likely.

Proposed Applications:
DNA origami structures could be used to interact with and stabilise **tight junction proteins** in the BBB, preventing the **leakage** of solutes. Additionally, interactions with membranes could be further used on the BBB endothelial cells, as DNA structures could carry materials to **promote the repair of damaged cells or membranes** (7)

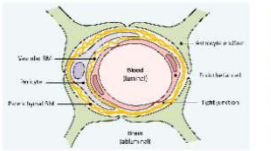

Alzheimer's Disease:
Alzheimer's disease (AD) is **the most common neurodegenerative disorder**, which manifests as memory impairment, cognitive dysfunction, personality changes and language disorders

Apoptotic neural death is an important cause of AD, however treatment is limited due to the BBB's regulation.
DNA nanotechnology is useful for precise medicine and could help to overcome the limitations of the BBB. **Tetrahedral DNA** (8) nanostructures (TDNs) which **inhibit the apoptosis of nerve cells** could be used to pass through the BBB, effectively inhibiting apoptosis and improving memory and learning ability. In certain concentrations these TDNs had no side effects on nerve cells and had a **simple synthesis** with a **high yield** and good **biocompatibility**. Therefore, along with manipulations of the membrane, this method could support nerve regeneration and limit the inflammatory responses of the BBB.

Social and Ethical Implications

'Currently the adoption of new technologies is accelerating and is occurring in a climate of suspicion and mistrust in medical evidence or reassurances.' - Paul Braveman, University of California.
Within DNA origami these misconceptions are amplified by the absence of research, and public general knowledge that DNA is specific to an individual so could be dangerous for various reasons.

We distributed a questionnaire to people of varying levels of knowledge in DNA origami:

- Over **50%** said they had never heard of DNA origami, although many could elaborate on uses of DNA in medicine
- However, on a scale of 1-10 of how far you agree, the statement **'DNA is a tool which may be dangerous if used incorrectly'** had an average of 6.95 (leaning towards agree).
- Whilst the statement **'DNA is a tool which may be used unethically'** had an average score of 7.29.
- When asked about negative ethical implications of DNA origami, many spoke about the 'misuse of personal information' and 'effects on future generations' despite the previous statement they knew little about the technology.
- Conversely, questions about the fears or concerns of DNA origami being used in medicine mainly said they would trust the research and approval of medical professionals.

Overall, it may be theorised in untrustworthy news articles that DNA origami may be used to the advantage of people wishing to take peoples identities, building on **misconceptions** and amplifying fears, as seen in previous releases of new drugs. Little to no evidence would be required to cause mass hysteria within 'clickbait' articles, and this could have a detrimental effect on the acceptance of these methods of treatment, whereas in reality, DNA Origami is an ethical solution to complex disorders. Despite public suspicion, a majority are likely to trust medical professionals.

Analysis & Conclusions

DNA origami has several potential uses within neuroscientific medicine due to its **high precision and controlled reconfigurable status**. Within delicate conditions such as epilepsy and Alzheimer's disease, DNA origami can support nerve stability and regeneration in response to certain signals, and modulate ion channels to prevent excessive neural activity. Public knowledge of DNA origami as a therapeutic treatment is low, and many expressed concern over the use of DNA in medicine, suggesting that wider education on the use of DNA nanostructures could be beneficial.

IRIS

References
1. Recent Advances in DNA Origami-Engineered Nanomaterials and Applications | Chemical Reviews (acs.org)
2. https://magazine.medlineplus.gov/article/understanding-different-kinds-of-seizures
3. Biomolecular mechanisms of epileptic seizures and epilepsy: a review | Acta Epileptologica | Full Text (biomedcentral.com)
4. https://www.ncbi.nlm.nih.gov/books/NBK2510/
5. Thought-Controlled Nanoscale Robots in a Living Host (plos.org)
6. (PDF) Drug Penetration into the Central Nervous System: Pharmacokinetic Concepts and In Vitro Model Systems (researchgate.net)
7. DNA origami scaffold promoting nerve guidance and regeneration - PubMed (nih.gov)
8. Cell Proliferation | Cell Biology Journal | Wiley Online Library

the BRAGG centre FOR MATERIALS RESEARCH

HENRY ROYCE INSTITUTE

DNA origami: Designing targeted treatments for bone marrow cancer

IRIS The Institute for Research in Schools

Summary
Using DNA Origami in creating a 3D structure, we have made a theoretical treatment for Bone Marrow Cancer. This aims to only have the drug attacked and received by cancerous cells instead of all the cells in the marrow. The structure is a cage-like structure that has the drug in the middle. We wanted to look into this area of DNA Origami due to the cancer's aggressive nature and since the life expectancy for the patient is only 5 years. We also noticed that other forms of treatment for bone marrow cancer tend to be harsh on the patients body leading to a longer recovery time and we want to reduce the recovery time so the patient's life can get back to normality

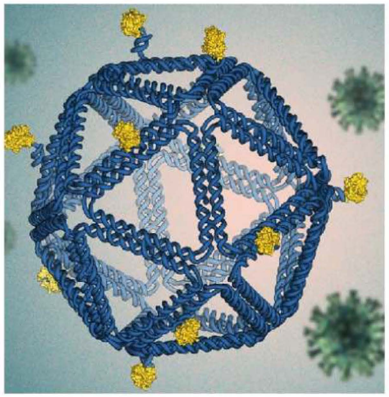

Engineers use "DNA origami" to identify vaccine design rules | MIT News | Massachusetts Institute of Technology

3D DNA Origami Structure
This Image above is show the initial idea for the cage shape so the drug is protected by the DNA and so the body is less likely to reject the structure since the DNA is similar to the DNA in the body. It will also be cheaper then having a solid shape. With Further research we would have looked at the cages for different shapes like cuboids or pentagonal and test it's strength and weakness. The diagram below shows what the structure will look like with the drug in it

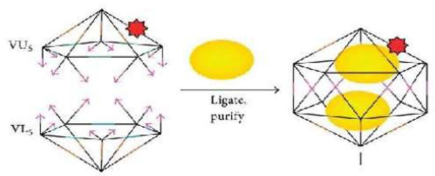

https://www.researchgate.net/figure/Cargo-molecules-are-passively-loaded-onto-3D-DNA-based-container-after-joining-the-two_fig12_276907662

Bone Marrow Cancer
The bone marrow is mainly organised by the bone, the vasculature and a network of reticular stromal cells. This diagram shows the structure of bone marrow:

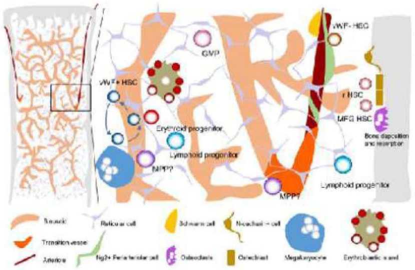

Bone marrow cancer is when bone marrow makes abnormal white cells know as leukaemia cells. Patients may experience a range of symptoms from pain in the location of the tumour, swelling or feeling lumps to difficulty moving. Some of the less common symptoms are weight loss, fevers fatigue and weakened bone. The life expectancy of someone with bone marrow cancer is 5 years with A 71% survival rate if found in the early stages.

Current Treatments
The treatments for bone marrow cancer are:

- Stem cell or bone marrow transplant which is from a donor
- High doses of chemotherapy and radiotherapy

Chemotherapy and radiotherapy are very harsh on the body since they kill all the cells in the area it is used on making the patients immune system weaker and more prone to illnesses and diseases

Theoretical Treatment
The treatment we have designed uses DNA origami to create a cage like structure to carry the drug to the cancerous cell. The drug we would be using is called Pomalidomide, which encourages the immune system to kill cancerous cells and block the development of new blood vessels which helps the cancer grow. However, this drug has the side effect of a low amount of white blood cells, skin sensitivity to light, inflammation to the bowel and sever changes in the kidney and liver. Using the 3D structure to carry the drug, it would prevent other cells being attacked by the drug and the patient recovery time would decrease. Since the patient would not have to undergo surgery to have a bone marrow transplant, the patient would be less prone to disease and illness as they would not be taking Ciclosporin so the body accept the new bone marrow

The theoretical treatment we have developed is derived from previous methods of treatment involving pomalidomide, a drug commonly used in cancer treatment which prevents and Stops the myeloma cancer cells from developing and also stops blood vessel growth which prevents the cancer cells growth as the vessels help them to survive. Pomalidomide also helps the immune system to kill myeloma cells.

Commonly used alongside Pomalidomide is Dexamethasone, a steroid, used to enhance the performance pomalidomide and aids it to work better at killing myeloma cells but us mostly used when someone has relapsed cancer. In general, the overall response rate of pomalidomide, for the use of killing myeloma cells, is 47% and the median survival is 13.9 months and in comparison to lenalidomide, another drug used in cancer treatment, pomalidomide is proven to be more potent.

The reason we have chosen pomalidomide to be used in our theoretical treatment is due to the fact that in comparison to other drugs, we have found it to be most suitable for bone marrow cancer as it helps the bone marrow to produce regular blood cells and the rate of response is relatively high. However there are some side effects to pomalidomide such as: body aches, chest pain, difficult breathing, dizziness and more. Overall, after considering the positives and negatives of pomalidomide we have decided to use it as the positives outweigh the negatives

Conclusion
In conclusion, we believe the cage structure of our DNA vessel is most effective as it protects the drug inside from any harm, it is cheaper to make in comparison to a solid spherical shape due to less DNA needed to make it and also is less likely to be rejected by the body because the DNA used to create the vessel is similar to the body DNA the body's natural immune system is less likely to detect the vessel as a foreign body allowing it to work effectively and efficiently in partnership with pomalidomide

L54

the BRAGG centre FOR MATERIALS RESEARCH
HENRY ROYCE INSTITUTE

Development of DNA Origami Nanostructures for Application in Medicine

IRIS The Institute for Research in Schools

REFERENCES

1. Chen X, Liu D, Wu Y, Yao H, Xia Q, Yang Y. Investigation of the Transporting Behavior of Framework DNA Nano-Devices Across the Artificial Blood-Brain Barrier (BBB). Chembiochem. 2022 Nov 4;23(21):e202200459. doi: 10.1002/cbic.202200459. Epub 2022 Oct 6. PMID: 36094759.
2. Ronca V, Wootton G, Milani C, Cain O. The Immunological Basis of Liver Allograft Rejection. Front Immunol. 2020 Sep 2;11:2155. doi: 10.3389/fimmu.2020.02155. PMID: 32983177; PMCID: PMC7492390.
3. Dean L. Blood Groups and Red Cell Antigens [Internet]. Bethesda (MD): National Center for Biotechnology Information (US); 2005. Chapter 5, The ABO blood group. Available from: https://www.ncbi.nlm.nih.gov/books/NBK2267/
4. Andersen, E., Dong, M., Nielsen, M. *et al.* Self-assembly of a nanoscale DNA box with a controllable lid. *Nature* 459, 73–76 (2009). https://doi.org/10.1038/nature07971
5. Veneziano, R., Moyer, T.J., Stone, M.B. *et al.* Role of nanoscale antigen organization on B-cell activation probed using DNA origami. *Nat. Nanotechnol.* 15, 716–723 (2020). https://doi.org/10.1038/s41565-020-0719-0
6. Rosier, B. J. H. M., Cremers, G. A. O., Engelen, W.,Merkx ,M., Brunsveld ,L., de Greef, T. F. A. .*et al.* Incorporation of native antibodies and Fc-fusion proteins on DNA nanostructures via a modular conjugation strategy 10.1039/C7CC04178K (Communication) *Chem. Commun.*, 2017, 53, 7393-7396

Overview

DNA Origami in Medicine

DNA origami has medical applications in targeted drug delivery, improving drug accuracy, and reducing toxicity. It can create containers for drug delivery to specific cells or tissues, improving drug pharmacokinetics, and enhancing efficacy. Additionally, it can be used for tumour imaging, monitoring cancer treatment efficacy, and as an alternative to traditional vaccines. In diagnostics, it can create probes for disease markers and point-of-care diagnostic devices.

Preliminary Ideas

Alzheimer's

DNA origami may bypass drug delivery challenges to the brain and restore neuronal function, with potential for treating cerebral issues. We proposed using DNA origami to carry enzymes that can break down NFTs across the BBB, based on previous research [1][4]. Figure 1 illustrates the enzyme containing DNA nanostructure. monomers.

Figure 1

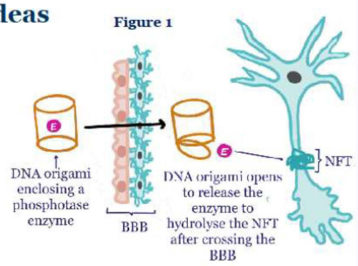

Dialysis

DNA origami technology could eliminate the need for dialysis or organ transplants by creating the filtration system for a biomechanical kidney implant (see figure 2). This implant would use nano-scale hexagonal plates (see figure 3) to tessellate to form a partially permeable membrane to filter the blood and would reduce infections and damage caused by current treatments.

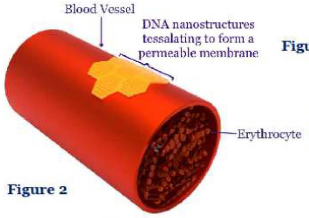

Figure 2

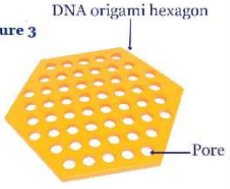

Figure 3

Cancer & Chemotherapy

Using DNA Origami, we could improve drug effectiveness by delivering it only to the tumour cells. The DNA origami mimics an antibody and has a complementary shape for antigens on tumour cells. The nanostructure could also be altered by an enzyme to potentially increase drug binding to the tumour. Figure 5 shows the catalytic action of the DNA nanostructure and enzyme as the removal of staples moves the drug closer to tumour cells.

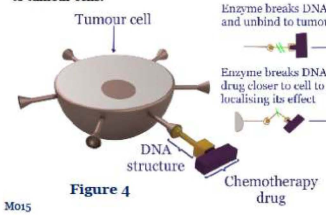

Figure 4

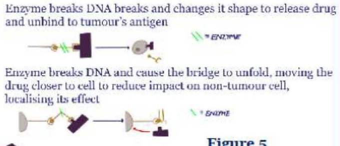

Figure 5

M015

Transplant + Rejection

DNA origami could solve compatibility issues by re-expressing matching antigenic surface glycoproteins and avoiding immune response as shown in Figure 6. This could reduce immunorejection and allow "universal" transplants, boosting transplantation rates and success.

Figure 6

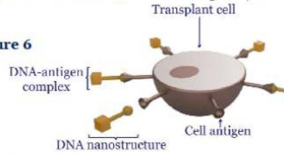

Final Design – RCAG

From the preliminary ideas, we decided to continue researching into transplantation and how we could apply DNA origami problems, focusing on primarily on how we could universalise donatory transplants, by re-expressing cell-surface antigenic glycoproteins (RCAG) to prevent rejection. However, it was determined that perfectly mimicking the binding site would not be possible with current design limitations, but it could be a potential area for future research.

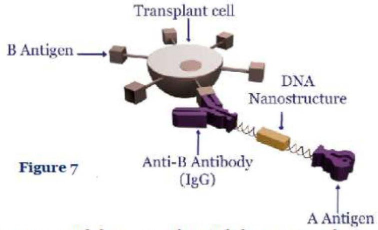

Figure 7

Therefore, we expanded our research to include a structure that would allow for an attachment site for the antigen/antibody. A published paper had done this research and stated that a protein could allow for the intermediary binding of antibodies to DNA nanostructures that is site-specific. It uses a protein that has a single-strand of DNA exposed - an oligodeoxynucleotide (ODN) exposed, enabling the nanostructure to bind to the protein through complementary base pairing of DNA. This gives us control over where the protein attaches to the nanostructure, and thus where the antibody binds. However, a different protein would have to be research for the attachment to the antigen due to its different chemical composition. It would also required an exposed ODN. This allows us to position the antigen and antibody geometrically opposite from each other, which facilitates our concept of re-expression of antigens.

Figure 8

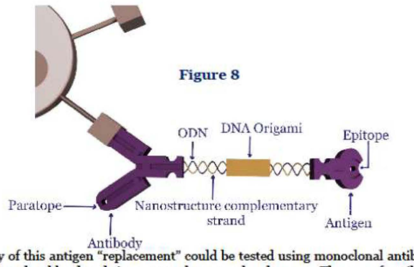

The efficacy of this antigen "replacement" could be tested using monoclonal antibodies to determine what blood and tissue type the organ has become. The use of antibodies for the antigen concealment could be replaced by aptamers, single stranded oligonucleotides that fold into defined structures and bind to targets similarly to antibodies. The aptamer would not require the conjugatory strategy as there is an immediate compatibility whereby aptamers may be attached to ODNs.

Integration of Software

Designing a Smiley Face in Scadnano

Our first major project on caDNAno single loop of scaffold and implement stapling for a complicated design, which posed a challenge due to the irregular shape requiring holes for the eyes and mouth. Issues with our stapling technique highlighted displacement between helices. We tried an alternative stapling method, which reduced fluctuations but did not eliminate distortion when viewed from the side.

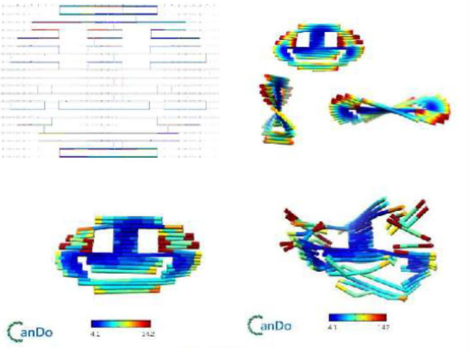

Figure 9

Designing a Simple Cube in caDNAno

For our first project in the migration to caDNAno, we began with a simple cube to get familiar with the software. We started with a row of helices spanning left to right, then alternating rows throughout the structure. This made adding crossovers easier on the 2D scaffold. We used CanDo to visualize helix strength, but its limited views were not sufficient. Switching to ChimeraX allowed us to produce a 3D model, but lacked strength testing. By combining the utilities of both the software, generating an analysis on CanDo and using a BILD file in ChimeraX, we were able to view the molecule's strength from all angles with the free view interface.

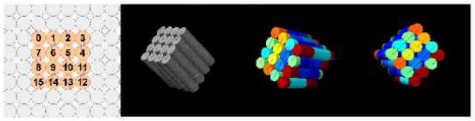

Figure 10

Final Design

The mechanism we designed to deliver our concept, for the re-expression of antigens on a cell, simply needed to connect an antigen on the cell surface to a different antigen of our choice, so there was little constraint in the structure we had to create. We settled on generating a tube-like structure to act as the connection piece between the antigens, as this provided simplicity and meant our molecule was sufficiently strong to prevent fluctuations, and theoretically when mass producing DNA origami to put our concept into practice a tube would be relatively quick and cost effective to produce.

Although it was not able to viewed on ChimeraX, our molecule had to contain single strands sticking out on either end as these provided connection points to the proteins, which in turn were bound to the antibody and antigen on either end; these are highlighted below on the 2D map of our design.

Final Design (Continued)

Our structure is essentially made by rolling the sheet up and wrapping it around itself to form two layers of DNA helices – as indicated by the order of the helices. The regular implementation of crossovers and stapling within the helices was achieved by using the auto-staple feature available within caDNAno, and then tweaking slightly to ensure length of staples were the correct length to bind the structure effectively. The inner ring of helices are also longer than the outer layer, as this provides a lot of central stability in the molecule while also providing almost a chamfered finish at the ends which allows greater range of access to the single strand connection point.

Figure 11

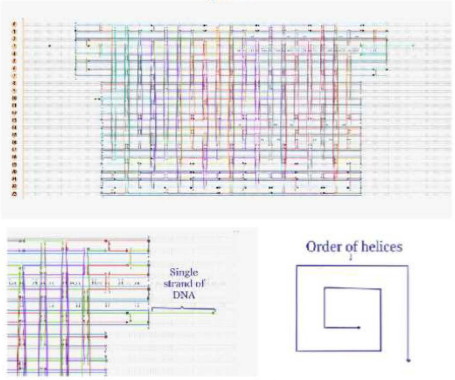

Visualisation of the Final Design

Once our structure had been created, we used CanDo to convert our JSON file to a BILD file, and viewed the results on ChimeraX:

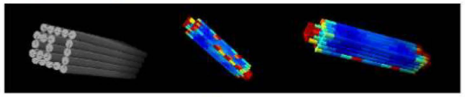

Figure 12

The molecule's length is substantial compared to its width, maintaining structural integrity especially in the central region, while weak spots were found at the ends, allow for flexibility in binding to the connection points. The molecule is slightly distorted down its length but this does not affect its function significantly.

Conclusion

The solution has potential however delivery inaccuracy requires excess DNA origami, which also need precise design to avoid warping, leading to a failure of re-expression and in succession, rejection. Further questions could arise in the aspect of cell growth and regeneration. The organ would be transplanted presenting, in theory, only compatible antigens but what happens when the cells that have the DNA origami bound to the antigens die? With more research, we could find potential solutions to these problems and relieve the tension on the donor system.

UNIVERSITY OF LEEDS
Bragg Centre for Materials Research

IRIS

DNA Origami
DNA based injectable hydrogels for tissue repair post ischaemic stroke

The Institute for Research in Schools

Ischaemic stroke: The issue

Stroke leads to irreparable brain tissue damage, creating an infarct site, consisting of the ischaemic core and penumbra. The ischaemic core renders permanent damage. The penumbra, however, is functionally compromised. Insufficient blood flow may cause necrosis, along with a multitude of other complications, due to the brain's ample oxygen/nutrient requirements.

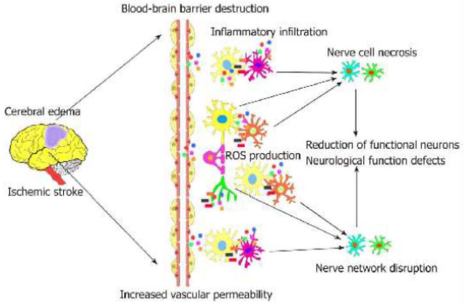

The possibility of neural stem cells

The underlying aim for stroke therapies are to restore damaged brain tissue. Utilising intracerebral stem cell transplantation is the foundation of strike therapy mechanisms, playing a vital role in neurogenesis, despite repair limitations.

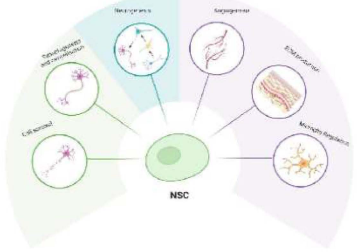

As a result, we set out to research and develop ways to utilise neuroplasticity to promote endogenous repair, as a regenerative technique for damaged brain tissue following ischaemic stroke.

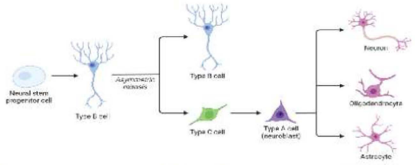

Issues caused by the extracellular matrix

Practical application of stem cell treatment is hindered due to lack of physical support in the extracellular matrix (ECM), leading to low cell viability, low proliferation and engraftment.

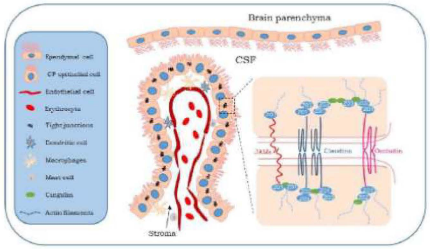

The microenvironment consisting of cerebrospinal fluid (CSF) circulates around ventricles and sub-arachnoid space, where it maintains brain homeostasis, therefore enhancing difficulty for stem cells to act effectively.

This can be counteracted via mimicry of the extracellular matrix with use of biomaterials, an example of which being hydrogels.

Solution: DNA Hydrogels

Hydrogels are hydrated scaffolds made up of cross-linked polymers. Since hydrogel fabrication can be performed using natural biopolymers (in this case, polynucleotides), scaffolds can mimic the ECM, often sharing mechanical and biochemical properties with the native tissue. These scaffolds will thus control the release of various cells and substances and offer physical support by providing a permissive environment for cell adhesion.

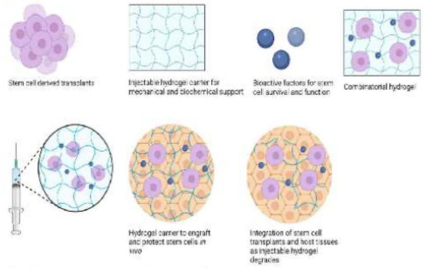

Polymerisation of molecules into networks entrap water molecules, which create water solubility. Hydrogels substitute the ECM lost after stroke, allowing cell adhesion, as adhesion to the ECM is vital in cell survival and tissue homeostasis.

Experimental Method

Scadnano software enabled us to create our own DNA hydrogel, comprised of a Y shaped scaffold made up of 3 ssDNA strands, each with a sticky end. There is also a linear linker molecule, made up of 2 ssDNA strands, each with a sticky end to attach to the Y scaffold.

Usual formation is via hydrogen bonding, physical entanglement or enzymatic reaction between chains. We focused primarily on utilising T4 ligase-catalysed reactions to form the cross-linked, polymeric structure of a hydrogel.

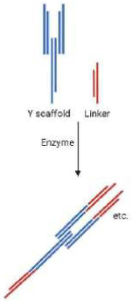

Results

The attempt to create our structure in scadnano rendered considerable complications, due to errors in the software. As displayed, the scaffold contained numerous sections of question marks extending far beyond the structure, along with ssDNA strands containing 0 bases.

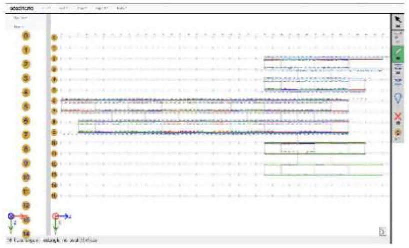

This meant our structure was unable to be exported in .csv format and therefore converted into a 3D image. This occurred in both the presence and absence of base deletions.

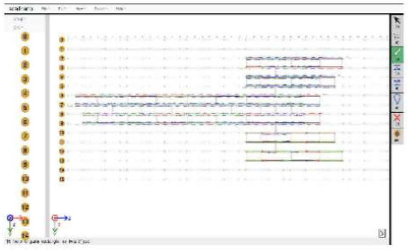

Conclusion

Despite software limitations which halted the final 3D render of our structure, we believe our research displays a promising application of DNA origami which has a fair possibility of success *in vivo*. Our next steps are to solve the issues relating to the modelling software so that the designs can be visualised and further refined.

the BRAGG centre FOR MATERIALS RESEARCH

HENRY ROYCE INSTITUTE
