# Supplementary material for: Science Outreach: Providing an Authentic Independent Research Opportunity in Materials Science to School Students
Source: J Chem Educ. 2026 May 18;103(6):2986–94. doi: 10.1021/acs.jchemed.5c00697 (PMC13261868; doi:10.1021/acs.jchemed.5c00697)
Supplement: Supplementary file 3 [file ed5c00697_si_003.pdf]

Science outreach: providing an authentic independent research opportunity in materials science to school students

Neil Garrido [a*], Andrew J. Lee [b], Clare Turnbull [a], Paolo Actis [b, c], Alison Rouncefield-Swales [a],

a Institute for Research in Schools, London, 165 Queen's Gate, London, SW7 5HD

b Bragg Centre for Materials Research, University of Leeds, Woodhouse Lane, Leeds, West Yorkshire, UK LS2 9JT

c School of Electronic and Electrical Engineering, University of Leeds, Woodhouse Lane, Leeds, LS2 9JT

The Institute for Research in Schools

DNA Origami Pilot Overview

Project Overview

In DNA origami students will learn how DNA can be used as a building material. The project starts off by introducing students to DNA nanotechnology and exploring the applications of this fascinating field of research. Through a number of tutorials, they will get to grips with caDNAno, a programme they will use to design their own nanostructure made from DNA.

The purpose of running this pilot project is to allow IRIS and the Bragg Centre to:

- evaluate and refine the project resources and materials which will be used in the final project when launched in September 2021
- evaluate student attitudes and perceptions to materials science

Project timeline

Like all IRIS projects, the DNA Origami Pilot follows a four-phase structure:

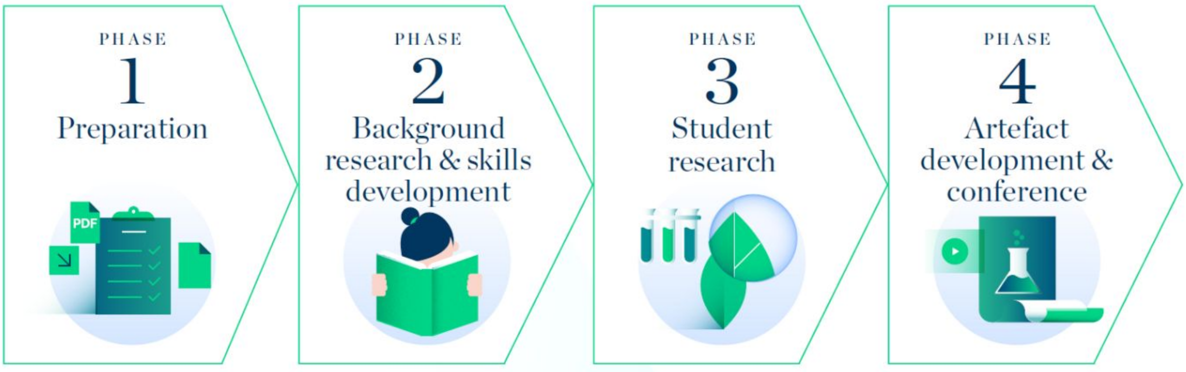

Phase 1: <u>Preparation</u>

- Select students
- Complete Avatar sheet* of students names and return to IRIS
- Organise installation of required software for DNA Origami (Python, caDNAno – see facilities and resources above)
- Complete teacher and student pre-participation survey
- Schedule weekly sessions to go through tutorial and exercises

* Avatar sheet is IRIS' school registration document

Phase 2: Background research and skill development

- Students will be expected to complete a series of online pre-recorded tutorials presented by [Bragg Centre academic]. The aim of these tutorials is to ensure students have the necessary skills and understanding of caDNAno to begin their own designs in **Phase 3**. Alongside the tutorials we have develop exercises to help students develop and refine their skills. These will be checked by IRIS and [Bragg Centre academic] and feedback provided. We encourage students to complete all tasks before moving on to the next tutorial.

- An outline of the tutorials is listed below:
  - **Tutorial 1: Scaffold DNA** – creating the template for DNA Origami
  - **Tutorial 2: Staples*** – creating the links to stablise the DNA Origami
  - **Tutorial 3: Calculating Sequences** – using caDNAno to calculate oligonucleotide strands
  - **Tutorial 4: CanDo** – evaluating stability of DNA Origami structures through computational analysis
  - **Tutorial 5: Beyond the basics** – a look at creating 3D DNA nanostructure designs

*Once tutorial 2 is completed, and they can successfully construct a suitable staple network, they will have sufficient skills to begin to work on their own designs. Please refer students to the background reading as well as suggested applications to help them make a start on their own designs.

Phase 3: Synthesis of DNA Origami

This phase is split into two parts: design and synthesise.

Part 1 - Design

Students will be **tasked with designing their own DNA origami structure**.

- Students are free to design their own shapes
- A limited number of applications will be provided to support any students who are unsure of what to design.
- Each design requires a rationale behind it and the designs should include some of the computational analysis to enable a discussion about the shapes stability.

Part 2 - Synthesise

Students will **synthesise a pre-planned DNA origami nanostructure**. All equipment and training will be provided. We will **liaise with individual schools prior to the shipment** of the chemicals and materials. The chemicals involved are low-hazard but should be stored in line with current health and safety legislation and school policy.

- Students carry out the synthesis of a pre-planned DNA nanostructure
- Sample to be returned to Bragg Centre, University of Leeds for analysis
- Visit to the Bragg Centre to meet [Bragg Centre academic] (or virtual meeting if necessary)
- Evaluation of DNA nanostructure using atomic force microscope
- Images of the sample will be sent to schools for use in phase 4

Phase 4: Artefact development and conference

Following the completion of Phase 3, students will create a research artefact – an academic poster, article, presentation, or academic paper. For this pilot project we would recommend that students focus on creating a research poster or presentation. A comprehensive guide on how to create an artefact will be provided to schools as well as support from your Regional School Engagement Lead. We would like schools taking part in the DNA Origami pilot to showcase their work at the IRIS Virtual Conference or at an online DNA Origami showcase.
