# Supplementary material for: Science Outreach: Providing an Authentic Independent Research Opportunity in Materials Science to School Students
Source: J Chem Educ. 2026 May 18;103(6):2986–94. doi: 10.1021/acs.jchemed.5c00697 (PMC13261868; doi:10.1021/acs.jchemed.5c00697)
Supplement: Supplementary file 5 [file ed5c00697_si_005.pdf]

Science outreach: providing an authentic independent research opportunity in materials science to school students

Neil Garrido [a]*, Andrew J. Lee [b], Clare Turnbull [a], Paolo Actis [b, c], Alison Rouncefield-Swales [a],

a Institute for Research in Schools, London, 165 Queen's Gate, London, SW7 5HD

b Bragg Centre for Materials Research, University of Leeds, Woodhouse Lane, Leeds, West Yorkshire, UK LS2 9JT

c School of Electronic and Electrical Engineering, University of Leeds, Woodhouse Lane, Leeds, LS2 9JT

DNA Origami Student Evaluation data

The following data was collected from students who participated in the DNA Origami project. Responses were gathered online at the end of the project or via a paper-based survey at conferences.

In the pilot year, the survey utilised an open and exploratory format which provided initial valuable insights but was unable to be robustly analysed. In 2022, IRIS enhanced our evaluation procedures by incorporating quantitative measures into our evaluation framework.

Table 1: Project participants by year

	**Pilot**	**2021-22**	**2022-23**	**2023-24**	**2024-25**
No. students	51	132	121	105	230
No. female	--	--	62	62	152
%age Female	--	--	51.2%	59.0%	66.1%

Table 2: Survey responses by year

	**Pilot and 2021-22**	**2022-23**	**2023-24**	**2024-25**
No. responses	19	22	38	50
No. female	--	10	17	33
%age Female	--	45.5%	44.7%	66.0%

Evaluation data from the pilot year

Table 3: Compared to **before** you took part in this project, how much more do you feel that you **now** know about…. [Response: Three-point Likert, Percentage based on student responding they know more]

	**Pilot year**
DNA nanotechnology	82.4%
Materials science	74.5%

Table 4: What were the best things about taking part in the DNA Origami

project? [Response: Single tick box]

	Pilot year
Using new software	75.0%
Having fun	75.0%
Learning about a topic outside the curriculum	68.8%
Working as a team	68.8%
Understanding more about science	68.8%
Contributing to science research	62.5%
Problem solving skills	62.5%
Working creatively	50.0%
Coming up with ideas	43.8%
Getting a better idea about studying science	43.8%
Making new friends	37.5%
Gaining confidence in public speaking	37.5%
Helping me identify a possible future career	31.3%
Learning about science careers	25.0%
Working independently	18.8%
Meeting scientists	18.8%
Making scientific discoveries	18.8%

Table 5: *Having taken part in the project, please indicate how far you agree with the following statements...* [Response options measured students' level of agreement with statements. Five-point Likert. Percentage based on student agreeing with the statement]

	Pilot year
I'm interested in doing independent research	81.3%
I found out more about possible careers	56.3%
I am more interested in a career in materials science	93.8%
I am more interested in a career in DNA nanotechnology	100.0%

Evaluation data from 2023 onwards

To provide a fuller picture of the project's impact, we have included summary data from the last two years of student surveys. This data demonstrates:

- high levels of student satisfaction.
- high proportions of students reporting improvement in key research skills and transferable skills.
- high proportions of students agreeing that they felt more confident in STEM, could make a valuable contribution to research, and understood how STEM can make a difference in the real world.
- increased knowledge of STEM careers, qualifications, and confidence about future study and career choices.

Table 6: How would you rate your experience of doing your IRIS project? [Response: Five-point Likert, Very poor - Very good]

	2023-24	2024-25
Total Student responses	38	50
No. rating 'good' or 'very good'	38	43
%age rating 'good' or 'very good'	100%	86.0%

Table 7: We would like to understand what impact working on your project has had on you and your skills, compared to before you started. How would you now rate your research skills? [Response options measured improvement in skills. 23-24: Three-point Likert; 24-25: Five-point Likert. Percentage based on student responding skills had improved]

	**2023-24**		**2024-25**	
	Male	Female	Male	Female
Formulating scientific questions	95.2%	81.3%	93.8%	69.7%
Working to a timescale or deadline	71.4%	58.8%	81.3%	75.8%
Finding information relevant to a topic	95.2%	94.1%	100%	97.0%
Evaluating sources of information	85.7%	94.1%	93.8%	84.8%
Analysing complex information	95.2%	87.5%	100%	87.9%
Understanding big data (added in 24 - 25)			56.3%	66.7%
Using IT to manage data / Using advanced IT or software (changed in 24 - 25)	71.4%	56.2%	75.0%	84.8%
Presenting your findings	100%	96.1%	81.3%	84.8%

Table 8: We would like to understand what impact working on your project has had on you and your skills, compared to before you started. How would you now rate your transferable skills? [Response options measured improvement in skills. 23-24: Three-point Likert; 24-25: Five-point Likert. Percentage based on student responding skills had improved]

	**2023-24**		**2024-25**	
	Male	Female	Male	Female
Speaking	76.2%	53.0%	87.5%	87.9%
Listening	95.2%	88.3%	87.5%	93.9%
Planning (23-24) Aiming High (24-25)	95.0%	82.4%	93.8%	87.9%
Staying positive (23-24) Adapting (24 - 25)	76.2%	76.5%	81.3%	75.0%
Creativity	95.2%	58.9%	93.8%	87.9%
Problem solving	100%	76.5%	100%	96.6%
Team working	90.5%	76.5%	86.7%	66.7%
Leadership	85.7%	52.9%	93.8%	69.7%

Table 9: Impact on self, identify and confidence: Has taking part in your IRIS project changed how you feel about the following? [Response options measured students' level of agreement with statements. 2022-23: Three-point Likert; 24-25: Five-point Likert. Percentage based on student agreeing with the statement]

	**2023-24**		**2024-25**	
	Male	Female	Male	Female
I am confident of my abilities in STEM	90.5%	88.2%	75.0%	69.7%
I am confident exploring ideas with other people			75.6%	75.8%
Other people think I am good at STEM			62.6%	63.7%

Table 10: Impact on understanding of STEM research: Has taking part in your IRIS project changed how you feel about the following? [Response options measured students' level of agreement with statements. 2022-23: Three-point Likert; 24-25: Five-point Likert. Percentage based on student agreeing with the statement]

	**2023-24**		**2024-25**	
	Male	Female	Male	Female
Research can be hard work but is rewarding	85.0%	94.1%	86.7%	90.7%
Success in research is a team effort	90.5%	76.5%	81.2%	94.0%

I can make a valuable contribution to research	85.0%	76.5%	87.5%	90.9%
I know about STEM beyond the classroom	95.2%	88.2%	93.7%	100%
I know how STEM can make a difference in the real world	100%	100%	93.7%	100%

Table 11: Impact on knowledge and understand of STEM jobs and careers: Has taking part in your IRIS project changed how you feel about the following? [Response options measured students' level of agreement with statements. 2022-23: Three-point Likert; 24-25: Five-point Likert. Percentage based on student agreeing with the statement]

	**2023-24**		**2024-25**	
	Male	Female	Male	Female
I know what it is like to work in STEM	81.0%	82.4%	75.0%	75.8%
People like me are researchers	85.0%	70.6%	80.0%	63.8%
I know about a range of different STEM careers	81.0%	96.1%	75.1%	84.8%
I am interested in a career in STEM	85.0%	82.4%	62.6%	75.8%
People like me work in STEM	90.0%	82.4%	75.0%	75.6%

Table 12: NEW STATEMENTS FOR 2024-25 Impact on knowledge and understand of STEM jobs and careers: Has taking part in your IRIS project changed how you feel about the following? [Response options measured students' level of agreement with statements. 24-25: Five-point Likert. Percentage based on student agreeing with the statement]

	**2024-25**	
	Male	Female
I know what qualifications I need to have a job related to STEM	87.6%	87.9%
Being a researcher would be an interesting career	80.0%	75.1%
I have developed important skills that will help me in the future	93.8%	94.0%
I better understand what it might be like to study STEM at a higher level	87.5%	97.0%
I feel confident about my future study and career choices	87.5%	71.9%
I have explored new areas of STEM	100%	93.9%
