# Supplementary material for: Science Outreach: Providing an Authentic Independent Research Opportunity in Materials Science to School Students
Source: J Chem Educ. 2026 May 18;103(6):2986–94. doi: 10.1021/acs.jchemed.5c00697 (PMC13261868; doi:10.1021/acs.jchemed.5c00697)
Supplement: Supplementary file 6 [file ed5c00697_si_006.docx]

Science outreach: providing an authentic independent research opportunity in materials science to school students

Neil Garrido ^a^*, Andrew J. Lee ^b^, Clare Turnbull ^a^, Paolo Actis ^b, c^, Alison Rouncefield-Swales ^a^,

a Institute for Research in Schools, London, 165 Queen’s Gate, London, SW7 5HD

b Bragg Centre for Materials Research, University of Leeds, Woodhouse Lane, Leeds, West Yorkshire, UK LS2 9JT

c School of Electronic and Electrical Engineering, University of Leeds, Woodhouse Lane, Leeds, LS2 9JT

**DNA Origami Student Evaluation data**

The following data was collected from students who participated in the DNA Origami project. Responses were gathered online at the end of the project or via a paper-based survey at conferences.

In the pilot year, the survey utilised an open and exploratory format which provided initial valuable insights but was unable to be robustly analysed. In 2022, IRIS enhanced our evaluation procedures by incorporating quantitative measures into our evaluation framework.

Table 1: Project participants by year

|  | Pilot | 2021-22 | 2022-23 | 2023-24 | 2024-25 |
| --- | --- | --- | --- | --- | --- |
| No. students | 51 | 132 | 121 | 105 | 230 |
| No. female | -- | -- | 62 | 62 | 152 |
| %age Female | -- | -- | 51.2% | 59.0% | 66.1% |

Table 2: Survey responses by year

|  | Pilot and 2021-22 | 2022-23 | 2023-24 | 2024-25 |
| --- | --- | --- | --- | --- |
| No. responses | 19 | 22 | 38 | 50 |
| No. female | -- | 10 | 17 | 33 |
| %age Female | -- | 45.5% | 44.7% | 66.0% |

**Evaluation data from the pilot year**

Table 3: Compared to **before** you took part in this project, how much more do you feel that you **now** know about.... [Response: Three-point Likert, Percentage based on student responding they know more]

|  | Pilot year |
| --- | --- |
| DNA nanotechnology | 82.4% |
| Materials science | 74.5% |

Table 4: What were the best things about taking part in the DNA Origami project? [Response: Single tick box]

|  | Pilot year |
| --- | --- |
| Using new software | 75.0% |
| Having fun | 75.0% |
| Learning about a topic outside the curriculum | 68.8% |
| Working as a team | 68.8% |
| Understanding more about science | 68.8% |
| Contributing to science research | 62.5% |
| Problem solving skills | 62.5% |
| Working creatively | 50.0% |
| Coming up with ideas | 43.8% |
| Getting a better idea about studying science | 43.8% |
| Making new friends | 37.5% |
| Gaining confidence in public speaking | 37.5% |
| Helping me identify a possible future career | 31.3% |
| Learning about science careers | 25.0% |
| Working independently | 18.8% |
| Meeting scientists | 18.8% |
| Making scientific discoveries | 18.8% |

Table 5: *Having taken part in the project, please indicate how far you agree with the following statements...* [Response options measured students’ level of agreement with statements. Five-point Likert. Percentage based on student agreeing with the statement]

|  | Pilot year |
| --- | --- |
| I'm interested in doing independent research | 81.3% |
| I found out more about possible careers | 56.3% |
| I am more interested in a career in materials science | 93.8% |
| I am more interested in a career in DNA nanotechnology | 100.0% |

**Evaluation data from 2023 onwards**

To provide a fuller picture of the project’s impact, we have included summary data from the last two years of student surveys. This data demonstrates:

- high levels of student satisfaction.
- high proportions of students reporting improvement in key research skills and transferable skills.
- high proportions of students agreeing that they felt more confident in STEM, could make a valuable contribution to research, and understood how STEM can make a difference in the real world.
- increased knowledge of STEM careers, qualifications, and confidence about future study and career choices.

Table 6: How would you rate your experience of doing your IRIS project? [Response: Five-point Likert, Very poor - Very good]

|  | 2023-24 | 2024-25 |
| --- | --- | --- |
| Total Student responses | 38 | 50 |
| No. rating ‘good’ or ‘very good’ | 38 | 43 |
| %age rating ‘good’ or ‘very good’ | 100% | 86.0% |

Table 7: We would like to understand what impact working on your project has had on you and your skills, compared to before you started. How would you now rate your research skills? [Response options measured improvement in skills. 23-24: Three-point Likert; 24-25: Five-point Likert. Percentage based on student responding skills had improved]

|  | 2023-24 | | 2024-25 | |
| --- | --- | --- | --- | --- |
|  | Male | Female | Male | Female |
| Formulating scientific questions | 95.2% | 81.3% | 93.8% | 69.7% |
| Working to a timescale or deadline | 71.4% | 58.8% | 81.3% | 75.8% |
| Finding information relevant to a topic | 95.2% | 94.1% | 100% | 97.0% |
| Evaluating sources of information | 85.7% | 94.1% | 93.8% | 84.8% |
| Analysing complex information | 95.2% | 87.5% | 100% | 87.9% |
| Understanding big data (added in 24 - 25) |  |  | 56.3% | 66.7% |
| Using IT to manage data / Using advanced IT or software (changed in 24 - 25) | 71.4% | 56.2% | 75.0% | 84.8% |
| Presenting your findings | 100% | 96.1% | 81.3% | 84.8% |

Table 8: We would like to understand what impact working on your project has had on you and your skills, compared to before you started. How would you now rate your transferable skills? [Response options measured improvement in skills. 23-24: Three-point Likert; 24-25: Five-point Likert. Percentage based on student responding skills had improved]

|  | 2023-24 | | 2024-25 | |
| --- | --- | --- | --- | --- |
|  | Male | Female | Male | Female |
| Speaking | 76.2% | 53.0% | 87.5% | 87.9% |
| Listening | 95.2% | 88.3% | 87.5% | 93.9% |
| Planning (23-24) Aiming High (24-25) | 95.0% | 82.4% | 93.8% | 87.9% |
| Staying positive (23-24) Adapting (24 - 25) | 76.2% | 76.5% | 81.3% | 75.0% |
| Creativity | 95.2% | 58.9% | 93.8% | 87.9% |
| Problem solving | 100% | 76.5% | 100% | 96.6% |
| Team working | 90.5% | 76.5% | 86.7% | 66.7% |
| Leadership | 85.7% | 52.9% | 93.8% | 69.7% |

Table 9: Impact on self, identify and confidence: Has taking part in your IRIS project changed how you feel about the following? [Response options measured students’ level of agreement with statements. 2022-23: Three-point Likert; 24-25: Five-point Likert. Percentage based on student agreeing with the statement]

|  | 2023-24 | | 2024-25 | |
| --- | --- | --- | --- | --- |
|  | Male | Female | Male | Female |
| I am confident of my abilities in STEM | 90.5% | 88.2% | 75.0% | 69.7% |
| I am confident exploring ideas with other people |  |  | 75.6% | 75.8% |
| Other people think I am good at STEM |  |  | 62.6% | 63.7% |

Table 10: Impact on understanding of STEM research: Has taking part in your IRIS project changed how you feel about the following? [Response options measured students’ level of agreement with statements. 2022-23: Three-point Likert; 24-25: Five-point Likert. Percentage based on student agreeing with the statement]

|  | 2023-24 | | 2024-25 | |
| --- | --- | --- | --- | --- |
|  | Male | Female | Male | Female |
| Research can be hard work but is rewarding | 85.0% | 94.1% | 86.7% | 90.7% |
| Success in research is a team effort | 90.5% | 76.5% | 81.2% | 94.0% |
| I can make a valuable contribution to research | 85.0% | 76.5% | 87.5% | 90.9% |
| I know about STEM beyond the classroom | 95.2% | 88.2% | 93.7% | 100% |
| I know how STEM can make a difference in the real world | 100% | 100% | 93.7% | 100% |

Table 11: Impact on knowledge and understand of STEM jobs and careers: Has taking part in your IRIS project changed how you feel about the following? [Response options measured students’ level of agreement with statements. 2022-23: Three-point Likert; 24-25: Five-point Likert. Percentage based on student agreeing with the statement]

|  | 2023-24 | | 2024-25 | |
| --- | --- | --- | --- | --- |
|  | Male | Female | Male | Female |
| I know what it is like to work in STEM | 81.0% | 82.4% | 75.0% | 75.8% |
| People like me are researchers | 85.0% | 70.6% | 80.0% | 63.8% |
| I know about a range of different STEM careers | 81.0% | 96.1% | 75.1% | 84.8% |
| I am interested in a career in STEM | 85.0% | 82.4% | 62.6% | 75.8% |
| People like me work in STEM | 90.0% | 82.4% | 75.0% | 75.6% |

Table 12: NEW STATEMENTS FOR 2024-25 Impact on knowledge and understand of STEM jobs and careers: Has taking part in your IRIS project changed how you feel about the following? [Response options measured students’ level of agreement with statements. 24-25: Five-point Likert. Percentage based on student agreeing with the statement]

|  | 2024-25 | |
| --- | --- | --- |
|  | Male | Female |
| I know what qualifications I need to have a job related to STEM | 87.6% | 87.9% |
| Being a researcher would be an interesting career | 80.0% | 75.1% |
| I have developed important skills that will help me in the future | 93.8% | 94.0% |
| I better understand what it might be like to study STEM at a higher level | 87.5% | 97.0% |
| I feel confident about my future study and career choices | 87.5% | 71.9% |
| I have explored new areas of STEM | 100% | 93.9% |
